# Dibasic Derivatives of Phenylcarbamic Acid against Mycobacterial Strains: Old Drugs and New Tricks?

**DOI:** 10.3390/molecules23102493

**Published:** 2018-09-28

**Authors:** Ivan Malík, Jozef Csöllei, Ivan Solovič, Šárka Pospíšilová, Hana Michnová, Josef Jampílek, Alois Čížek, Iva Kapustíková, Jana Čurillová, Mária Pecháčová, Jiřina Stolaříková, Daniel Pecher, Michal Oravec

**Affiliations:** 1 Department of Pharmaceutical Chemistry, Faculty of Pharmacy, Comenius University in Bratislava, Odbojárov 10, SK-832 32 Bratislava, Slovakia; sharka.pospisilova@gmail.com (Š.P.); michnova.hana@gmail.com (H.M.); josef.jampilek@gmail.com (J.J.); kapustikova@fpharm.uniba.sk (I.K.); curilova2@uniba.sk (J.Č.); mariapech901@gmail.com (M.P.); 2 Department of Chemical Drugs, Faculty of Pharmacy, University of Veterinary and Pharmaceutical Sciences in Brno, Palackého 1946/1, CZ-612 42 Brno, Czech Republic; csolleij@vfu.cz; 3 Clinic for Tuberculosis and Lung Diseases, National Institute for Tuberculosis, Lung Diseases and Thoracic Surgery, Vyšné Hágy, SK-059 84 Vysoké Tatry, Slovakia; solovic@hagy.sk or ivan.solovic@ku.sk; 4 Department of Public Health, Faculty of Health, Catholic University in Ružomberok, Hrabovská cesta 1A, SK-034 01 Ružomberok, Slovakia; 5 Clinic for Department of Infectious Diseases and Microbiology, Faculty of Veterinary Medicine, University of Veterinary and Pharmaceutical Sciences, Palackého 1946/1, CZ-612 42 Brno, Czech Republic; cizeka@vfu.cz; 6 Laboratory for Mycobacterial Diagnostics and Tuberculosis, Regional Institute of Public Health, Partyzánské náměstí 7, CZ-702 00 Ostrava, Czech Republic; Jirina.Stolarikova@zu.cz; 7 Department of Pharmaceutical Analysis and Nuclear Pharmacy, Faculty of Pharmacy, Comenius University in Bratislava, Odbojárov 10, SK-832 32 Bratislava, Slovakia; pecher1@uniba.sk; 8 Toxicological and Antidoping Center, Faculty of Pharmacy, Comenius University in Bratislava, Odbojárov 10, SK-832 32 Bratislava, Slovakia; 9 Global Change Research Institute CAS, Belidla 986/4a, CZ-603 00 Brno, Czech Republic; oravec.m@czechglobe.cz

**Keywords:** dibasic phenylcarbamates, surface tension, electronic properties, lipophilicity, *Mycobacterium* spp.

## Abstract

In order to provide a more detailed view on the structure–antimycobacterial activity relationship (*SAR*) of phenylcarbamic acid derivatives containing two centers of protonation, 1-[2-[({[2-/3-(alkoxy)phenyl]amino}carbonyl)oxy]-3-(dipropylammonio)propyl]pyrrolidinium oxalates (**1a**–**d**)/dichlorides (**1e**–**h**) as well as 1-[2-[({[2-/3-(alkoxy)phenyl]amino}carbonyl)oxy]-3-(di-propylammonio)propyl]azepanium oxalates (**1i**–**l**)/dichlorides (**1m**–**p**; alkoxy = butoxy to heptyloxy) were physicochemically characterized by estimation of their surface tension (γ; Traube’s stalagmometric method), electronic features (log *ε*; UV/Vis spectrophotometry) and lipophilic properties (log *k*_w_; isocratic RP-HPLC) as well. The experimental log *k*_w_ dataset was studied together with computational logarithms of partition coefficients (log *P*) generated by various methods based mainly on atomic or combined atomic and fragmental principles. Similarities and differences between the experimental and *in silico* lipophilicity descriptors were analyzed by unscaled principal component analysis (PCA). The *in vitro* activity of compounds **1a**–**p** was inspected against *Mycobacterium tuberculosis* CNCTC My 331/88 (identical with H_37_R_v_ and ATCC 2794, respectively), *M.* *tuberculosis* H_37_R_a_ ATCC 25177, *M.* *kansasii* CNCTC My 235/80 (identical with ATCC 12478), the *M.* *kansasii* 6509/96 clinical isolate, *M.* *kansasii* DSM 44162, *M. avium* CNCTC My 330/80 (identical with ATCC 25291), *M.* *smegmatis* ATCC 700084 and *M. marinum* CAMP 5644, respectively. *In vitro* susceptibility of the mycobacteria to reference drugs isoniazid, ethambutol, ofloxacin or ciprofloxacin was tested as well. A very unique aspect of the research was that many compounds from the set **1a**–**p** were highly efficient almost against all tested mycobacteria. The most promising derivatives showed *MIC* values varied from 1.9 μM to 8 μM, which were lower compared to those of used standards, especially if concerning ability to fight *M.* *tuberculosis* H_37_R_a_ ATCC 25177, *M.* *kansasii* DSM 44162 or *M. avium* CNCTC My 330/80. Current *in vitro* biological assays and systematic *SAR* studies based on PCA approach as well as fitting procedures, which were supported by relevant statistical descriptors, proved that the compounds **1a**–**p** represented a very promising molecular framework for development of ‘non-traditional’ but effective antimycobacterial agents.

## 1. Introduction

**Figure 1:**
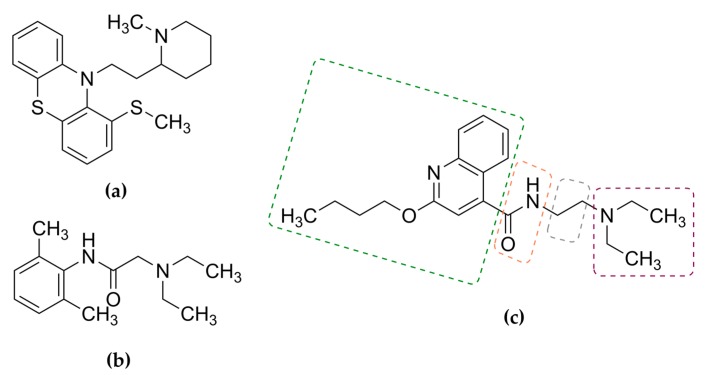
Chemical structure of thioridazine (**a**), a neuroleptic drug containing a phenothiazine scaffold (**a**); as well as effective local anesthetics (**b**) lidocaine (xylocaine); and (**c**) dibucaine (cinchocaine). All these compounds showed notable *in vitro* efficiency against some mycobacterial strains [5,6,7,8,9,10,11,12]. Structural fragments of dibucaine were color-coded as follows: a lipophilic moiety (a dark green framework), polar group (orange), connecting hydrocarbon chain (gray) and salt-forming (basic) group (dark red), respectively.

The treatment of commonly encountered species of tuberculous and non-tuberculous mycobacteria responsible for a multiplicity of different types of infections, including pulmonary, respiratory, cutaneous, and systemic infections, by (i) brand new classes of promising compounds preferably acting on novel targets; or (ii) ‘non-typical’ antimycobacterial drug candidates is still in very dynamic and progressive debate [1,2,3,4,5,6]. The strategy was successfully used in a case of *in vitro* screening of some β-lactam antibiotics (ceftaroline or ceftazidime) in a combination with an β-lactamase inhibitor avibactam against *Mycobacterium avium* complex [3,4]. Another encouraged example was *in vitro* and *ex vivo* testing of tricyclic thioridazine (Figure 1a), an old neuroleptic phenothiazine. The molecule was used alone or in a combinatorial therapy with anti-tuberculosis drugs (isoniazid, rifampin, linezolid or moxifloxacin) against *M. tuberculosis* regardless of its antibiotic resistance phenotype and was highly active due to its multi mechanisms of action [5,6].

Antimycobacterial properties of compounds originally designed and tested as local anesthetics (LAs) have been investigated sporadically. Moreover, their application to management of the infections caused by a broad spectrum of species of the *Mycobacterium* genus has not been systematically reviewed. Nevertheless, Schmidt and Rosenkranz [7] as well as Fujii et al. [8] found that lidocaine (xylocaine; Figure 1b), a highly efficient LA drug [9,10], notably inhibited *in vitro* growth of some strains of human *M. tuberculosis*, *M. bovis* or *M. kansasii*. Similarly, a quinoline core-containing dibucaine (cinchocaine; Figure 1c) [11], was able to effectively *in vitro* fight rapidly growing *M. phlei* [12].

A common structural feature of given efficient LAs (and promising antimycobacterials) was presence of a lipophilic moiety, polar anilido or amido group, connecting hydrocarbon chain and salt-forming fragment. All considered moieties were color-differentiated for a molecule of dibucaine (cinchocaine; Figure 1c).

Sequencing of *Mycobacterium* spp. genomes proved that these microorganisms produced a variety of enzymes [13,14,15] able to hydrolyze ester, anilide or amide bonds in a chemical structure of antimycobacterial drug candidates. Isosteric replacement of these polar groups with a carbamate moiety would provide very good proteolytic stability, especially if the modified structural arrangement of novel derivatives would be as follows: Aryl–NH–C(O)O–Alkyl [16]. The carbamate functionality imposes a degree of conformational restriction due to delocalization of non-bonded electrons on nitrogen into a carboxyl moiety. Participation of the carbamate group in hydrogen bonding through carboxyl and backbone N–H [17] should be also taken into account. Considering the importance of the carbamate functionality, its incorporation into a structure of novel antimycobacterial agents was one of key tasks in a process of their design and synthesis [18,19,20].

**Scheme 1:**
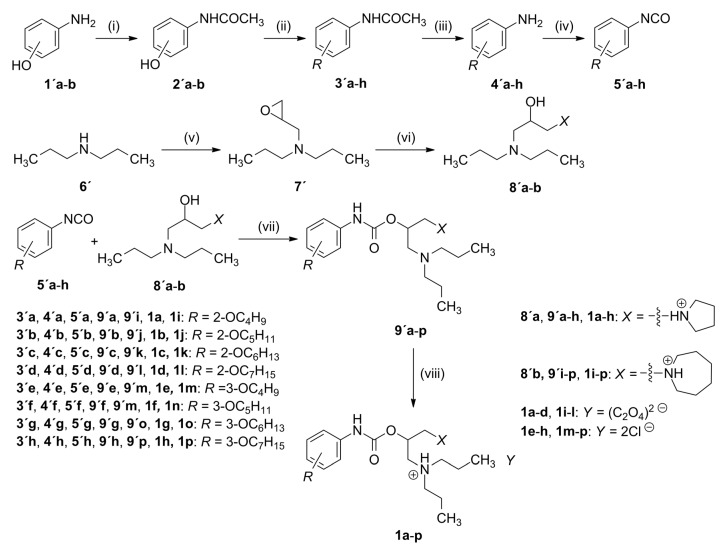
Synthesis of 1-[2-[({[2-/3-(alkoxy)phenyl]amino}carbonyl)oxy]-3-(dipropylammonio)-propyl]pyrrolidinium/azepanium oxalates/dichlorides (**1a**–**p**; alkoxy = butoxy to heptyloxy). *Reagents and conditions*: (i) (CH_3_CO)_2_O, reflux (1h); (ii) Na, anhydrous EtOH, 1-bromoalkane (alkane = butane to heptane), r.t. (12h), reflux (3h); (iii) 18% HCl, reflux (2h); (iv) phosgene, anhydrous toluene, reflux (3h); (v) (±)-2-(chloromethyl)oxirane, 35 °C (2h), r.t. (48h), 38% NaOH; (vi) pyrrolidine/azepane, anhydrous 2-PrOH, reflux (6h); (vii) anhydrous toluene, reflux (8h); (viii) saturated solution of (a) oxalic acid in anhydrous EtOH or (b) ethereal hydrogen chloride.

Currently evaluated 1-[2-[({[2-/3-(alkoxy)phenyl]amino}carbonyl)oxy]-3-(dipropylammonio)-propyl]pyrrolidinium/azepanium oxalates/dichlorides (**1a**–**p**; alkoxy = butoxy to heptyloxy; Table 1, Scheme 1) were synthesized with intention to find effective LAs with favorable toxicity profiles [21]. The compounds contained two types of an anionic counterpart (**1a**–**d** and **1i**–**l** versus **1e**–**h** and **1m**–**p**; Table 1) in order to achieve convenient aqueous solubility in biological assays [21].

It was found that all tested derivatives showed higher indices defining their relative surface (*U*_s_) as well as infiltration (*U*_i_) local anesthetic efficiency than reference LA drugs cocaine (*U*_s_ = 1.0, *U*_i_ = 3.6) or procaine (*U*_s_ = 0.1, *U*_i_ = 1.0) [21]. The most effective dibasic molecule in given types of local anesthesia was 1-[2-[({[3-(pentyloxy)phenyl]amino}carbonyl)oxy]-3-(dipropylammonio)propyl]-pyrrolidinium dichloride (**1f**) with *U*_s_ = 130 and *U*_i_ = 250 (Table S1 in Supplementary Materials), respectively.

It might be expected that the carbamate moiety was sterically protected due to branching of both connecting hydrocarbon chain and salt-forming group. Rotatable bonds of the 2-/3-alkoxy side chain *R* could also contribute to the steric hindrance (Table 1). It was proved by *ab initio* procedures that an intramolecular hydrogen bond between N–H of the carbamate functionality and oxygen atom of the 2-alkoxy chain was formed [22]. Such structural arrangements of compounds would provide stability against effects of various mycobacterial enzymes and would consider them promising candidates for antimycobacterial agents.

It was observed that surface tension (relative surface activity), electronic and lipophilic properties of carbamate group-containing molecules notably influenced the *in vitro* antimycobacterial efficiency [23,24,25]. However, the set **1a**–**p** has not been satisfactorily characterized by values of relevant physicochemical descriptors [26] (Table S1).

In a first part of this study, attention was turned on more precise physicochemical characterization of the derivatives **1a**–**p**. The research aimed determination of compounds’ ability to decrease surface tension of water (γ), estimation of electronic properties described by logarithms of molar absorption coefficients (log *ε*) of their methanolic solutions, which were investigated in the UV/Vis region of an electromagnetic spectrum, as well as evaluation of lipophilic features defined by calculated log *k*_w_ parameters. These log *k*_w_ were based on extrapolation procedures of estimated logarithms of retention factors (log *k*) to elution with 100% water by reversed-phase high-performance liquid chromatography (RP-HPLC). In addition, relationships between the log *k*_w_ dataset and calculated logarithms of partition coefficients (log *P*) related to the octan-1-ol/water partitioning system were explored in order to provide a critical view on possibilities to predict lipophilic properties of the compounds based on two-dimensional (2D) visualization of their chemical structures.

Next, a very essential objective of the research was *in vitro* screening of the derivatives **1a**–**p** against various strains of tuberculous and non-tuberculous mycobacteria, i.e., *M. tuberculosis* CNCTC My 331/88, *M. tuberculosis* H_37_R_a_ ATCC 25177, *M. kansasii* CNCTC My 235/80, a *M. kansasii* 6509/96 clinical isolate, *M. kansasii* DSM 44162, *M. avium* CNCTC My 330/80, *M. smegmatis* ATCC 700084 and *M. marinum* CAMP 5644, respectively.

A key aim of the study was to find some structural and physicochemical features of the compounds **1a**–**p**, which might appear to be notable for their *in vitro* antimycobacterial efficiency.

## 2. Results and Discussion

### 2.1. Synthesis of the Compounds ***1a**–**p***

The investigated compounds **1a**–**p** were prepared by multi step pathways using 2-aminophenol (**1′a**) and 3-aminophenol (**1′b**), respectively, as starting molecules (Scheme 1). Very briefly, a reaction of **1′a** or **1′b** with acetanhydride led to *N*-(2-/3-hydroxyphenyl)ethanamide (**2′a** or **2′b**). A solution of **2′a** or **2′b** in anhydrous ethanol (EtOH) was added to sodium ethanoate. After mixing, 1-bromoalkane (alkane = butane to heptane) was added in order to prepare a series of *N*-(2-/3-alk-oxyphenyl)ethanamides (**3′a**–**h**; alkoxy = butoxy to heptyloxy). The molecules **3′a**–**h** were suspended in 18% hydrochloric acid and heated up to reflux. The solutions were cooled, neutralized and crude intermediates were extracted into diethyl ether (DEE). The organic layer was dried, filtered and solutions were removed *in vacuo* giving 2-/3-alkoxyanilines (**4′a**–**h**; alkoxy = butoxy to heptyloxy) [27]. Reulting intermediates **4′a**–**h** were dissolved in anhydrous toluene and added continuously into a saturated solution of phosgene [27]. The mixed solutions were refluxed, toluene was removed *in vacuo* providing desired 1-alkoxy-2-/3-isocyanatobenzenes (**5′a**–**h**).

Into an aqueous solution of *N*-propylpropanamine (**6′**), a (±)-2-(chloromethyl)oxirane reagent was added and solution was allowed to stand at room temperature (r.t.; 48h). The mixture was heated up to 75 °C and treated with 38% sodium hydroxide. The cooled solution was filtered and a crude intermediate was formed. Extraction of the filtrate with DEE, drying and removal of the organic layer provided additional amount of the intermediate [28], which isolation and crystallization from absolute EtOH led to (±)-*N*-(oxiran-2-ylmethyl)-*N*-propylpropanamine (**7′**).

The addition of pyrrolidine or azepane to **7′** in anhydrous propan-2-ol (2-PrOH) provided 1-(dipropylamino)-3-pyrrolidin-1-ylpropan-2-ol (**8′a**) or 1-azepan-1-yl-3-(dipropylamino)-propan-2-ol (**8′b**) [29].

Oily 1-(1-azacycloalkyl)-3-(dipropylamino)propan-2-yl (2-/3-alkoxyphenyl)carbamates (**9′a**–**p**; azacycloalkyl = pyrrolidinyl or azepanyl) were prepared by a reaction of 1-alkoxy-2-/3-isocyanato-benzenes **5′a**–**h** with a dibasic alcohol **8′a** or **8′b** in anhydrous toluene.

Addition of a saturated solution of oxalic acid in anhydrous EtOH or ethereal hydrogen chloride to the bases **9′a**–**p** dissolved in chloroform led to solid colorless 1-[2-[({[2-/3-(alkoxy)phenyl]- amino}carbonyl)oxy]-3-(dipropylammonio)propyl]pyrrolidinium oxalates (**1a**–**d**)/dichlorides (**1e**–**h**) and 1-[2-[({[2-/3-(alkoxy)phenyl]amino}carbonyl)oxy]-3-(dipropylammonio)propyl]azepanium oxalates (**1i**–**l**)/dichlorides (**1m**–**p**; Scheme 1, Table 1) as well. More detailed procedures describing syntheses of particular intermediates and final molecules were provided in Supplementary Materials.

Chemical structure of the compounds **9′a**–**p** and **1a**–**p** was previously verified by their infrared (IR) spectra, which confirmed presence of all key groups. In addition, elemental analyses results (% C, H, N) were within ±0.40% of theoretical values for both bases **9′a**–**p** and salts **1a**–**p** [21]. Melting points (m.p.**′**s), *R*_f_ values (TLC) and acid-base p*K*_a1_ and p*K*_a2_ parameters of **1a**–**p** were already published [21,26] and can be found in Table S1.

Current liquid chromatography high resolution mass spectroscopy (HPLC-HR-MS) analyses, which were performed on the LC Agilent Infinity System coupled with the Quadrupole Time-Of-Flight Mass Spectrometer (6520 Accurate Mass Q-TOF LC/MS), confirmed structural identity of the intermediates **9′a**–**p** (Supplementary Materials).

After re-crystallization from a mixture of acetone/ethanol (subgroups **1a**–**d, 1e**–**h** and **1m**–**p**) or acetone (**1i**–**l**), high-resolution mass spectra (HR-MS) of these salts were measured by the Dionex UltiMate 3000 High-Performance Liquid Chromatograph coupled with the LTQ Orbitrap XL Hybrid Ion Trap-Orbitrap Fourier Transform Mass Spectrometer equipped with a HESI II (heated electrospray ionization) source in a positive (**1a**–**d**, **1i**–**l**) or negative (**1e**–**h**, **1m**–**p**) mode. The analyses confirmed structural identity of given molecules.

### 2.2. Determination and Prediction of Some Physicochemical Properties of the Compounds ***1a**–**p***

#### 2.2.1. Surface Tension

Surface tension (relative surface activity; γ) of aqueous solutions of the compounds **1a**–**p** (*c* = 2.0 × 10^−3^ M) was determined by a drop count technique [30,31] using a Traube stalagmometer. All evaluated substances were able to decrease surface tension of water (γ = 0.07259 N/m) at 21 °C.

**Figure 2:**
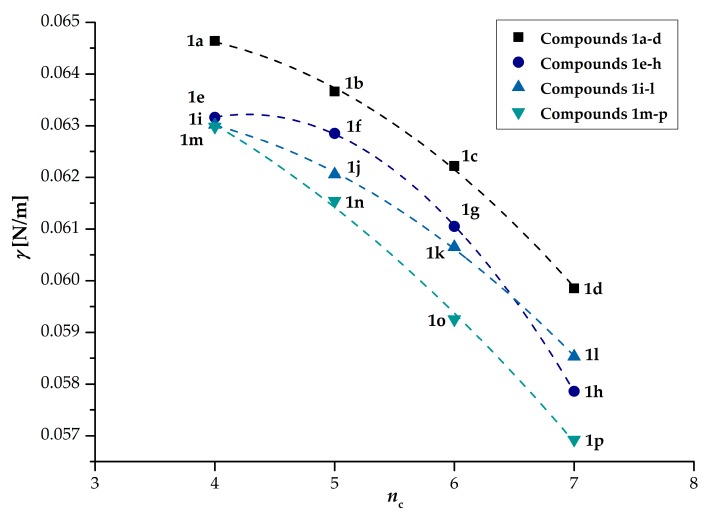
Relationships between number of carbon atoms forming the 2-/3-alkoxy side chain *R* (*n*_c_; alkoxy = butoxy to heptyloxy) and γ values (in N/m units) of the compounds **1a**–**p**.

Elongation of the 2-/3-alkoxy side chain *R* led to more surface-active derivatives within homological sets **1a**–**d**, **1e**–**h**, **1i**–**l** and **1m**–**p**. This behavior was also in agreement with conclusions of a research paper [32]. The 2-alkoxy substituted molecules **1a**–**d** and **1i**–**l** showed lower ability to reduce surface tension of water than their 3-alkoxy substituted isomers **1e**–**h** or **1m**–**p** (Table 1, Figure 2).

Relationships between the number of carbon atoms forming the chain *R* (*n*_c_) and γ values (in N/m units) was inspected using a linear function, polynomial function of 2^nd^ order and sigmoidal fitting, respectively.

Equations related to the most relevant functions and values of common statistical descriptors, namely, number of points (number of cases; *n*), degrees of freedom (*DF*), reduced chi-square (*χ*,^2^_red_), residual sum of squares (*RSS*), correlation coefficient (*R*), adjusted coefficient of determination (*Adj. R*^2^), root mean squared error (standard deviation; *RMSE*), norm of residuals (*NR*), Fisher’s significance ratio (Fisher’s *F*-test; *F*) and probability of obtaining the *F* Ratio (significance of a whole model; *Prob > F*), respectively, can be found in Table S2.

Indication of a significance level of the *F* Ratio was as follows: one star symbol (*) for statistically significant, two stars symbol (**) for statistically very significant or three stars symbol (***) for statistically extremely significant level was used. The regression equations and their statistical characteristics were calculated and visualized by the Origin Pro *ver.* 9.0.0 SR2 software (OriginLab Corporation, Northampton, MA, USA).

The relationships between *n*_c_ and γ were described most precisely by statistically significant (**1a**–**d**, **1i**–**l** and **1m**–**p**) or very significant (**1e**–**h**) models built on polynomial functions of 2nd order (Figure 2, Equations (S1–S4) in Table S2).

The derivatives containing an azepanium moiety (**1i**–**l**, **1m**–**p**) showed slightly higher ability to decrease surface tension of water than their positional isomers with a pyrrolidinium salt-forming group (**1a**–**d**, **1e**–**h**). The most surface active were 1-[2-[({[3-(heptyloxy)phenyl]amino}carbonyl)-oxy]-3-(dipropylammonio)propyl]azepanium dichloride (**1p**) with γ = 0.05692 N/m and 1-[2-[({[3-(heptyloxy)phenyl]amino}carbonyl)oxy]-3-(dipropylammonio)propyl]pyrrolidinium dichloride (**1h**), which showed γ = 0.05786 N/m (Table 1, Figure 2).

**Figure 3:**
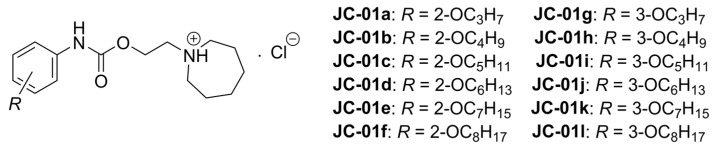
Chemical structure of the compounds **JC-01a**–**l**, effective local anesthetics [33], which were able to decrease surface tension of water [34].

Very similar behaviors to present findings were observed when Čižmárik with co-workers [33,34] analyzed structurally similar compounds **JC-01a**–**l**, effective LAs [33], which contained only one centrum of basicity (Figure 3). All given molecules showed ability to decrease surface tension of water and 3-alkoxy substituted derivatives **JC-01g**–**l** (alkoxy = propoxy to octyloxy) were more efficient surfactants than their 2-alkoxy positional isomers **JC-01a**–**f**. Relationships between *n*_c_ (*n*_c_ = 3–8) and γ (in N/m units) of these alkoxy positional isomers were described very satisfactorily by polynomial functions of 2nd order [34].

Concerning presently analyzed dichlorides **1m**–**p**, branching of their connecting chain and presence of a dipropylammonium moiety resulted in the γ values (Table 1), which were slightly lower in comparison to those of **JC-01h**–**k** [34].

#### 2.2.2. Electronic Properties

Electronic properties of the molecules **1a**–**p** (Table 1) were characterized by logarithms of molar absorption coefficients (log *ε*) of their methanolic solutions (*c* = 8.0 × 10^−5^ M) investigated in the UV/Vis region of an electromagnetic spectrum.

The solutions showed three absorption maxima in a near ultraviolet (quarz) region of this spectrum between 200 nm and 400 nm [35], namely, *λ*_1_ = 208–210 nm, *λ*_2 (Ch-T)_ = 236–238 nm and *λ*_3_ = 278–280 nm (Table 1), respectively. Positions of these absorption bands in the spectrum were typical for derivatives of (substituted) phenylcarbamic acid and the bands were assigned as first local excitation (*λ*_3_), charge-transfer (*λ*_2 (Ch-T)_) and second local excitation (*λ*_1_) absorption maxima, respectively [36]. The *λ*_1_ and *λ*_2 (Ch-T)_ maxima of 2-alkoxy substituted compounds **1a**–**d** and **1i**–**l** were slightly lower compared to those of their 3-alkoxy substituted positional isomers **1e**–**h** and **1m**–**p** (Table 1), respectively. Chemical structures of analyzed derivatives **1a**–**d** and **1i**–**l** indicated that steric inability of both carbamoyloxy and alkoxy groups to achieve coplanarity inhibited ‘proper’ resonance.

The log *ε*_2 (Ch-T)_ parameters of compounds **1a**–**p**, which were observed at *λ*_2 (Ch-T)_, varied from 4.01 (**1o**) to 4.52 (**1c**). The methanolic solutions of **1a**–**h** were characterized by higher log *ε*_2 (Ch-T)_ values than those of **1i**–**p** and ranged from 4.05 (**1b**) to 4.52 (**1c**; Table 1), excluding the molecules **1j** (4.22) and **1l** (4.14). In addition, there was found nor linear neither quasi-parabolic relationship between number of carbon atoms forming the side chain *R* (*n*_c_) and log *ε*_2 (Ch-T)_ values (Figure S1 in Supplementary Materials).

In next sections of the paper, which are aimed at relationships between structure and *in vitro* activity, special consideration is devoted to the log *ε*_2 (Ch-T)_ values because they could be the most sensitive to differences in electronic environment of a phenylcarbamoyloxy moiety due to different position and length of a substituent *R* [36].

#### 2.2.3. Lipohydrophilic Properties

Lipophilicity has been the physicochemical parameter of notable importance in *QSAR* and *SAR* studies as a predominant descriptor encoding information on a network of inter- and intramolecular forces affecting drug transport *via* lipophilic compartments as well as drug’s interactions with target effector sites [37,38].

A classical shake-flask method for a partitioning measurement of a compound in the octan-1-ol/buffer (or water) system, thus estimation of its log *P*_exp_ value, is quite time-consuming, it requires the solute to be pure and with adequate solubility in an aqueous phase. Insolubility often means that highly lipophilic compounds may not be determined accurately [39]. Concerning these issues very seriously, RP-HPLC was rather applied to estimate lipophilic properties of the molecules **1a**–**p** than the shake-flask alternative.

Octadecyl-functionalized silica gel was currently used as a stationary phase (SPh) and a gradient of two solvents at different volume ratios modified retention properties of the SPh [39,40]. Liquid binary mixtures of methanol (MeOH) with water were employed as mobile phases (MPhs) in the isocratic RP-HPLC. The MeOH modifier was preferred because of similarity of both observed and extrapolated lipophilicity parameters with the ones determined in the octan-1-ol/water partition system, considering also sensitivity to *H*-bond donor properties of investigated compounds [41].

The modifier was applied in different volume concentrations, which varied from 85% to 100% (*v*/*v*) with 5% increments. This interval was chosen due to very high lipophilicity of investigated salts. Practically, lower volume concentration of MeOH, i.e., less than 85% (*v*/*v*) in used MPhs, caused uncertainty of retention times (*t*_r_) due to notable peaks broadening, especially within subsets **1i**–**l** and **1m**–**p**. In addition, their *t*_r_ values were considerably prolonged with increase in water content (*v*/*v*) of the MPhs so it was no longer possible to determine *t*_r_ values in such MPhs. Taking the 85–100% volume concentrations range, the isocratic separation was feasible and reasonable retention of the compounds **1a**–**p** was observed in all MPhs. The highest *t_r_* parameters were found for the molecules **1k** (*t*_r_ = 47.108 min) and **1l** (*t*_r_ = 70.012 min) in the MPh containing 85% proportion (*v*/*v*) of MeOH (Table 2).

In addition, purity (in percentages) of the compounds **1a**–**p** was verified by RP-HPLC. Areas of their peaks were measured using the MPh, which contained 90% proportion (*v*/*v*) of MeOH. The purity of given salts varied from 96.82% (**1j**) to 99.65% (**1m**; Table S3).

Estimated retention factor (capacity factor; *k*) values were found in an area from 0.5063 (**1e**) to 2.5096 (**1l**) if pure MeOH was used as the MPh, and from 3.6174 (**1e**) to 30.4159 (**1l**) if the 80:20 MeOH/water (*v*/*v*) MPh was applied (Table S3).

Regarding a group of oxalates (Scheme 1), a pyrrolidium moiety-containing derivatives **1a**–**d** showed lower log *k* values in all used MPhs than those with an azepanium fragment **1i**–**l**. Same trends were observed for dichlorides, i.e., the compounds **1e**–**h** showed lower log *k* outputs compared to those of a series **1m**–**p**.

Elongation of the *R* substituent (Table 1) led to increase in *t*_r_ as well as log *k* values within all evaluated subgroups **1a**–**d**, **1e**–**h**, **1i**–**l** and **1m**–**p** (Table 2). Increase in a volume concentration (*v*/*v*) of MeOH caused shortening of both *t*_r_ and log *k* for all analyzed compounds **1a**–**p** (Table 2).

El Tayar et al. [42,43] investigated lipophilic properties of protonated basic compounds, which were therapeutically used as psychoactive agents, and inspected relationships between their log *k* values and volume concentrations (*v*/*v*) of MeOH. They found that these relationships were notably influenced by nature of solutes. The models were preferably built on linear functions and MeOH exerted its own solvophobic effect in the MPhs, which contained more than 80% (*v*/*v*) of given organic modifier if focusing on analyses of neutral and non-ionic compounds.

For partially and completely ionized polar compounds, the relationships appeared to be nearly parabolic but the parts of parabolic curves, which corresponded to MeOH-rich eluents (80% or higher volume concentration (*v*/*v*) of MeOH) were regarded as linear [42,43].

Paschke et al. [44] analyzed a series of highly lipophilic tetrachlorobenzyltoluene isomers by isocratic RP-HPLC and found linear relationships (*R* > 0.9995) between their log *k* values and volume concentrations of MeOH, which varied from 80% to 100% (*v*/*v*).

On the other hand, observed log *k* data could not be used in terms of a universal scale to express lipophilicity of compounds because the log *k* values were dependent on chromatographic conditions, i.e., they depended on both MPhs and SPhs. Extrapolation of estimated solute retention (log *k*) to elution with 100% water (calculation of log *k*_w_) would definitely be much more precise approach than using of log *k*, because the *k*_w_ parameters were independent of any effect of the organic modifier and relied on the SPh alone [45]. It has been recognized that the log *k*_w_ descriptor was very efficient for anticipation of *in vitro* antimycobacterial properties of compounds [46,47,48,49].

According to the authors’ opinions based on systematic scientific literature survey, a current research paper was the first, which aimed the log *k*_w_ parameters of 2-/3-alkoxyphenylcarbamic acid derivatives containing two centers of protonation. The log *k*_w_ values were extrapolated from intercepts of a relationship between log *k* and volume fraction of the MPh modifier (*ϕ*_M_) using the Snyder–Soczewiński linear solvent strength model [50,51,52]. The relationship was justified by *R* > 0.9900, *Adj. R*^2^ > 0.9750 as well as *F* > 180.00 for calculation of particular log *k*_w_ parameters (Table 3).

Goodness of fit for all proposed models generating log *k*_w_, in other words, quantity used to test whether any given data were well described by a suggested function, was adjusted by *χ*^2^_red_ values, which varied from 0.0002 (**1c**) to 0.0045 (**1o**). As can be seen, the linear relationships minimized *RSS* values, as variabilities about regression lines, which ranged from 0.0007 (**1c**) to 0.0134 (**1o**). The linearity was also proved by calculated *RMSE* parameters, as standard deviations of the data about regression lines, which were found in an interval from 0.0152 (**1c**) to 0.0668 (**1o**; Table 3).

The extrapolated log *k*_w_ values of compounds **1a**–**p** (Table 3) were in accordance with their elution order and hydrophobicity and varied from 3.7688 (**1a**) to 6.1749 (**1p**). Assuming presence of an identical azacycloalkyl moiety, oxalates (**1a**–**d**, **1i**–**l**) were less lipophilic than corresponding positional isomers synthesized as dichlorides (**1e**–**h**, **1m**–**p**; Table 3). As expected, 1-[2-[({[3-(hept-yloxy)phenyl]amino}carbonyl)oxy]-3-(dipropylammonio)propyl]azepanium oxalate (**1l**) and 1-[2-[({[3-(heptyloxy)phenyl]amino}carbonyl)oxy]-3-(dipropylammonio)propyl]azepanium dichloride (**1p**; Table 1) were found to be the most lipophilic showing log *k*_w_ of 5.8966 (**1l**) and 6.1749 (**1p**; Table 3), respectively.

Increase in *n*_c_ (Table 1) led to higher log *k*_w_ values in all inspected subgroups **1a**–**d**, **1e**–**h**, **1i**–**l** and **1m**–**p**. These relationships were defined by statistically significant (**1e**–**h**, **1i**–**l**), very significant (**1a**–**d**) and extremely significant (**1m**–**p**) linear functions and values of relevant statistical descriptors (Equations (S5)–(S8) in Table S4).

The *S* index (Table 3) in RP-HPLC has been a function of molecular structure parameters. For sets of non-polar homologues, linear relationships between their *S* and number of carbon atoms in a hydrocarbon side chain was found. Increase in size and van der Waals volume of a solute led to increased *S* parameter as well [53].

Results of present analyses agreed with given conclusions. Increase in length of the side chain *R* led to higher *S* within all inspected homological subgroups. The compounds containing an azepanium moiety showed higher *S* parameters, which varied from 4.3102 (**1i**) to 6.2672 (**1p**), than those with a pyrrolidium group, i.e., from 3.7768 (**1a**) to 5.6830 (**1h**; Table 3), if same position and length of *R* was presumed.

The slope *S* values of linear regression functions used to obtain log *k*_w_ were connected with specific hydrophobic surface areas of compounds and could serve as indicative measure of uniformity of retention mechanisms. If the uniformity was observed, a convenient model between the slope(s) and intercept(s), i.e., log *k*_w_ values, was anticipated [40]. Statistically extremely significant relationship between the log *k*_w_ and *S* values of **1a**–**p** was found and described by Equation (1):

*S* = 0.9449 (±0.0823) × log *k*_w_ + 0.2755 (±0.4087) (1)

*n* = 16, *DF* = 14, *χ*,^2^_red_ = 0.0470, *RSS* = 0.6586, *R* = 0.9508, *Adj. R*^2^ = 0.8971, *RMSE* = 0.2169, *NR* = 0.8115, *F* = 131.75, *Prob > F* = 0.0001 ***

Based on values of statistical descriptors provided above, the uniformity of a retention mechanism was proven and suitability of chosen MPhs was confirmed for lipophilicity evaluation of the derivatives **1a**–**p**.

Statistical analyses indicated that both position and length of the side chain *R* (Equations (S9) and (S10) in Table S5) were more important factors, which contributed to the uniformity of retention behavior, compared to nature (and physicochemical properties) of a salt-forming fragment (Equations (S11) and (S12) in Table S5) of inspected compounds, despite the fact that all partial models built on linear regression analyses were statistically extremely significant.

To provide better understanding and give a critical review of relationships between the experimental lipophilicity measures and *in silico* prediction of lipophilic properties, the log *k*_w_ dataset was studied together with computational logarithms of partition coefficients (log *P*).

Practical estimation of log *P*_exp_ for some molecules from the set **1a**–**p** by a shake-flask method was not possible due to their insolubility in phosphate buffer systems (p*H* = 7.30). Thus, the lack of enough reliable log *P*_exp_ values would be a very limiting factor for eventual investigation of the relationships between log *P*_exp_ and *in silico* log *P*. On the other hand, linear models between log *k*_w_ and log *P*_exp_ were already observed [39], so interchangeability of these descriptors was fully justified.

The log *P* parameters of non-protonated forms of analyzed compounds, i.e., **9′a**–**p** (Scheme 1, Table 4), were calculated for the octan-1-ol/water partitioning system. These values were generated by the ChemBioDraw Ultra 12.0 software package (CambridgeSoft, Cambridge, MA, USA), various Java and non-Java interactive applets as well.

The Ghose and Crippen′s log *P*_Cr_ [54,55], Viswanadhan′s log *P*_V_ [56], Broto’s log *P*_B_ [57] and Leo’s CLOGP 4.0 [58] atomic and atomic/fragmental-based approaches were chosen to calculate the log *p* values (Table 4) by the ChemBioDraw Ultra 12.0 software, respectively. The Virtual Computer Chemistry Laboratory [59], a freely available web-based tool working in Java environment, was used to calculate log *P* by the Wang’s XLOGP 2.0 [60], Cheng’s XLOGP 3.0 [61], Moriguchi’s MLOGP [62], Sander’s (Actelion’s) ACLOGP [63], Molinspiration’s miLogP 2.2 [64], ALOGP [65] and Tetko’s ALOGPs 2.1 [66] method (Table 4 and Table 5), respectively. All these methods integrated algorithms combining atomic and fragmental principles, except for ALOGPs 2.1, which considered a molecule in the whole [66].

Finally, the SILICOS-IT hybrid method, which used proprietary fragment- and property-based principles, was also employed to generate log *P* parameters (log *P*_S-IT_; Table 5). The method was implemented in the SwissADME applet, a free web tool designed to evaluate pharmacokinetics and drug-likeness of small molecules [67].

A common issue connected with given *in silico* approaches [26] was that they did not allow to correctly predict log *p* values of particular salts **1a**–**p**. This was the reason why current research targeted on the calculations related to non-protonated bases **9′a**–**p**.

The compounds **9′i**–**p** showed higher log *P* than their positional isomers **9′a**–**h**, which contained a different salt-forming moiety. Almost all used predictor tools indicated the molecules **9′a**–**p** highly lipophilic with log *p* > 4.27 (Table 4 and Table 5), excluding the MLOGP method. The calculations based on MLOGP indicated moderate or high lipophilicity of these substances, which log *P* were found in a range from 2.75 (**9′a** and **9′e**) to 3.76 (**9′l** and **9′p**; Table 5).

The CLOGP 4.0 predicted the highest lipophilic nature of the bases **9′a**–**p** and their log *P* varied from 6.23 (**9′a** and **9′e**) to 8.94 (**9′l** and **9′p**; Table 4). As might be assumed, all employed atomic and atomic/fragmental methods did not consider a position of the side chain *R* when generating log *P* (Table 4 and Table 5). Only the whole-molecule ALOGPs 2.1 approach took into account this aspect, so the 2-alkoxy substituted compounds **9′a**–**d** and **9′i**–**l** were less lipophilic than their 3-alkoxy substituted positional isomers **9′e**–**h** and **9′m**–**p** (Table 5), respectively.

It was found that linear relationships between the extrapolated log *k*_w_ values of compounds **1a**–**p** (Table 3) and particular *in silico* log *P* parameters of non-protonated derivatives **9′a**–**p**, namely, log *P*_Cr_, log *P*_V_, log *P*_B_, CLOGP 4.0, XLOGP 2.0, XLOGP 3.0, MLOGP, ACLOGP, miLogP 2.2, ALOGP, log *P*_S-IT_ and ALOGPs 2.1 (Table 4 and Table 5), respectively, were statistically extremely significant (*Prob > F* = 0.0001) and were defined by *R* > 0.9300, *Adj. R*^2^ > 0.8650, *F* > 99.00 and other common statistical descriptors (Equations (S13)–(S24) in Table S6).

In an effort to characterize these relationships more precisely, similarities and differences between given descriptors were analyzed by using unscaled principal component analysis (PCA), a powerful multivariate statistical technique, that analyzed a set of values, in which observations were described by several inter-correlated quantitative dependent variables. A set of new orthogonal variables, called principal components (PCs), and pattern of similarity of observations was a result the analysis [68].

Current PCAs were performed by the Origin Pro *ver.* 9.0.0 SR2 software. Number of concerned PCs was determined by visual evaluation of a scree plot, as a relationship between calculated eigenvalues (*λ*_e_) and number of PCs. The *λ*_e_ descriptor measured amount of variation retained by each PC [68]. The first two PCs, i.e., Principal Component 1 (PC 1) and Principal Component 2 (PC 2), of the analysis accounted for 99.26% of the total variance in the data. It meant that other PCs might be ignored without losing any substantial information. The PCs did not prove existence of ‘real’ parameters, they only indicated that existence of these descriptors was mathematically possible within the set of analyzed values.

**Figure 4:**
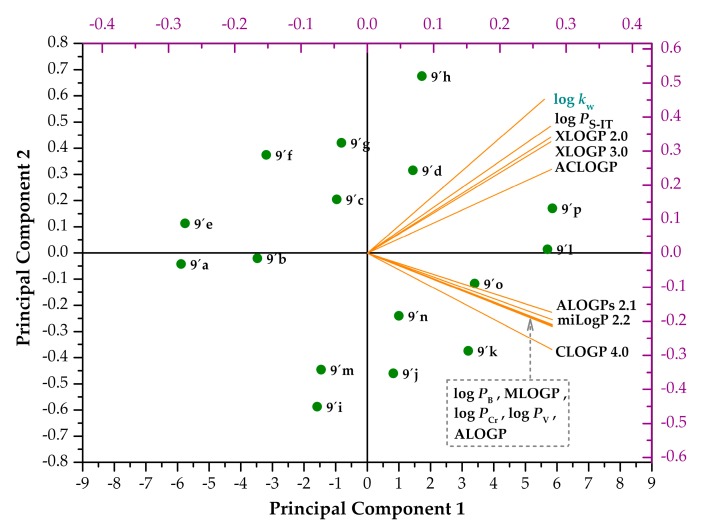
Two-dimensional biplot (mapping) showing both Principal Component 1 and 2 scores of the compounds **9′a**–**p** and loadings of extrapolated chromatographic (log *k*_w_; **1a**–**p**) and *in silico* lipophilicity variables, i.e., log *P*_S-IT_, XLOGP 2.0, XLOGP 3.0, ACLOGP, ALOGPs 2.1, miLogP 2.2, CLOGP 4.0, log *P*_Cr_, log *P*_V_, log *P*_B_, MLOGP and ALOGP, respectively.

A relationship between PC 1 and PC 2 resulted in division of examined compounds into characteristic groups. Differences in lipophilic properties of the bases **9′a**–**p** were reflected in their PC 1 values. One group, defined by negative PC 1 values, included derivatives containing a pyrrolidin-1-yl moiety (**9′a**–**c**, **9′e**–**g**) as well as molecules with both azepan-1-yl fragment and butoxy side chain (**9′i**, **9′m**). Conversely, second group, which was characterized by positive PC 1 values, encompassed compounds containing an azepan-1-yl moiety (**9′j**–**l**, **9′n**–**p**) as well as derivatives with both sterically smaller azacycloalkyl ring and heptyloxy substituent (**9′d**, **9′h**). The most negative PC 1 value was generated for the least lipophilic derivative **9′a**, which salt **1a** showed the lowest experimental lipophilicity (log *k*_w_ = 3.7688). The most positive PC 1 output was registered for the most lipophilic compound **9′p**, a base of the most lipophilic salt **1p** (log *k*_w_ = 6.1749; Figure 4).

It was found that PC 2 took into consideration a type of an azacycloalkyl moiety as well as position and length of *R*. The derivatives **9′a**–**h** showed higher PC 2 than the compounds **9′i**–**p**. Higher PC 2 were assigned to the 3-alkoxy substituted molecules compared to their 2-alkoxy positional isomers, when assuming their identical salt-forming moiety.

Increase in *n*_c_ of the side chain *R* led to higher PC 1 as well as PC 2. There were found no compounds (points), which could be regarded as outliers to the remaining ones along PC 1 or PC 2 (Figure 4).

The PCA-based 2D biplot showed both PC scores (PC 1, PC 2) of evaluated compounds (green dots) and loadings of variables indicated as orange straight lines [68]. The lines characterizing log *k*_w_, log *P*_I-IT_, XLOGP 2.0, XLOGP 3.0 and ACLOGP variables were located on upper right side of this biplot. The line related to log *k*_w_ formed the smallest angles with those of log *P*_I-IT_, XLOGP 2.0 or XLOGP 3.0, which meant that these descriptors correlated each other most positively. Conversely, the largest angle was observed between the lines connected with log *k*_w_ and CLOGP 4.0, so these variables were unlikely to correlate each other (Figure 4).

The PC 1 was influenced almost equally by loadings of all inspected variables, as indicated particular scores on top axis. The scores varied from 0.27 (log *k*_w_) to 0.28 (log *P*_Cr_, log *P*_V_, log *P*_B_, miLogP 2.2 and ALOGP, respectively). The most positive impact on PC 2 showed a loading of the log *k*_w_ and log *P*_I-IT_ variable, which scored approximately 0.45 and 0.37, respectively, on right axis. Conversely, the strongest inverse relationship was found between PC 2 and loading of the CLOGP 4.0 variable (−0.28; Figure 4).

Following visualization, the *in silico* log *P*_I-IT_, XLOGP 2.0 and XLOGP 3.0 descriptors might be used to characterize lipophilic properties of analyzed salts and bases, especially if they contained highly lipophilic side chain *R*. Those molecules were defined by positive values of both PC 1 and PC 2. In addition, very close grouping was observed for **9′l** with **9′p** (Figure 4).

Considering observed physicochemical characteristics of the compounds **1a**–**p**, there was found statistically extremely significant linear relationship between the γ and log *k*_w_ values. This dependence was expressed by Equation (2) and values of relevant statistical descriptors as follows:

γ = −0.0031 (±0.0003) × log *k*_w_ + 0.0765 (±0.0015) (2)

*n* = 16, *DF* = 14, *χ*,^2^_red_ = 6.436 × 10^−7^, *RSS* = 9.010 × 10^−6^, *R* = 0.9383, *Adj. R*^2^ = 0.8718, *RMSE* = 8.022 × 10^−4^, *NR* = 0.0030, *F* = 103.02, *Prob > F* = 0.0001 ***

Linearity of this model was adjusted by relatively very low *χ*^2^_red_, *RSS* and *RMSE* values. The γ and log *k*_w_ parameters of **1i**–**p** showed the most positive impact to final statistical analysis data (Equation (S28) in Table S7) connected with Equation (2) compared to those of a subset **1a**–**h** (Equation (S27) in Table S7).

One of the major tasks to consider in drug design is the ability of a compound to cross biological membranes. The blood–brain barrier (*BBB*) is a critical biological structure for maintaining homeostasis of central nervous system and preventing damage to the brain. Compounds primarily designed as neuroactive agents are required to cross the *BBB* to provide their therapeutic effect. Conversely, molecules that target other compartments of a body ideally should not cross this barrier to avoid possible psychotropic side effects. Thus, the task of predicting the *BBB* permeability of new compounds is of great importance [69].

Norinder and Haeberlein [70] suggested some simple rules to preliminary answer a question whether a drug candidate would be able to cross the *BBB*. Very briefly, if a sum of nitrogen and oxygen atoms (*n*_N_ + *n*_O_) in a molecule was ≤ 5 or if [CLOGP − (*n*_N_ + *n*_O_)] > 0 then the compound would have a good chance to enter the brain by passive diffusion. The originally employed CLOGP was a lipophilicity descriptor generated *in silico*. In addition, molecular weight (MW) of such compound should be ≤ 450 to facilitate brain permeation [71].

Each of the oxalates **1a**–**d** and **1i**–**l** contained (*n*_N_ + *n*_O_) = 10, each of the chlorides **1e**–**h** and **1m**–**p** contained (*n*_N_ + *n*_O_) = 6 (Table 1), respectively. If presently calculated CLOGP 4.0 parameter would be replaced with log *P*_S-IT_ (Table 5), which was closer to a ‘real’ log *k*_w_ lipophilicity descriptor (Figure 4), only basic forms of the most lipophilic compounds **1l** and **1p**, i.e., the substances **9′l** and **9′p**, might be considered to possess very limited brain permeation. On the other hand, calculated MW > 450 for these derivatives [21] indicated that they might not be able to passively cross the *BBB*.

### 2.3. In Vitro Antimycobacterial Assays

The *in vitro* activity of compounds **1a**–**p** was inspected against *Mycobacterium tuberculosis* CNCTC My 331/88 (identical with H_37_R_v_ and ATCC 2794, respectively; abbreviation used: *MT*_v_ H_37_R_v_), *M. tuberculosis* H_37_R_a_ ATCC 25177 (*MT*_a_ H_37_R_a_), *M. kansasii* CNCTC My 235/80 (identical with ATCC 12478; *MK* 235/80), a *M. kansasii* 6509/96 clinical isolate (*MK* 6509/96), *M. kansasii* DSM 44162 (*MK* DSM), *M. avium* CNCTC My 330/80 (identical with ATCC 25291; *MA* 330/80), *M. smegmatis* ATCC 700084 (*MS*) and *M. marinum* CAMP 5644 (*MM*), respectively, by the methods described earlier [20,72,73,74,75]. *In vitro* susceptibitity of given mycobacteria to reference drugs isoniazid (INH), ethambutol (EMB), ofloxacin (OFLX) or ciprofloxacin (CPX) was tested as well (Table 6 and Table 7).

A value of a minimum inhibitory concentration (*MIC*) was the lowest concentration of a tested compound, (i) which inhibited growth of *MT*_v_ H_37_R_v_, *MK* 235/80*, MK* 6509/96 and *MA* 330/80, respectively; (ii) at which no visible growth of *MT*_a_ H_37_R_a_, *MS*, *MK* DSM as well as *MM* was observed [20,72,73,74,75].

The *in vitro* screening procedures related to particular mycobacterial strains were repeated three times and the *MIC* values, reported in Table 6 and Table 7 (in μM units), were average values of these determinations.

A very unique aspect of a present research was that many compounds from the set **1a**–**p** were *in vitro* efficient against almost all tested mycobacteria. The most promising molecules with observed *MIC* ≤ 8 μM were indicated in gray (Table 6 and Table 7).

The 3-alkoxy substituted derivatives **1e**–**h** and **1m**–**p** were slightly more effective against all screened strains than their 2-alkoxy substituted isomers **1a**–**d** and **1i**–**l**, respectively. 1-[2-[({[3-(Heptyloxy)phenyl]amino}carbonyl)oxy]-3-(dipropylammonio)propyl]azepanium dichloride (**1p**) was the most active against almost complete spectrum of tested microorganisms (*MIC* ≤ 8 μM), excluding *MA* 330/80 only. Comparable efficiency to **1p** was observed for 1-[2-[({[3-(heptyl-oxy)phenyl]amino}carbonyl)oxy]-3-(dipropylammonio)propyl]pyrrolidinium dichloride (**1h**), which also showed *MIC* ≤ 8 μM against almost all tested mycobacteria, excluding *MA* 330/80 as well as *MS*. In addition, antimycobacterial activity of 1-[2-[({[3-(pentyloxy)phenyl]amino}carbonyl)oxy]-3-(di-propylammonio)propyl]azepanium dichloride (**1n**) and 1-[2-[({[3-(hexyloxy)phenyl]amino}-carbonyl)oxy]-3-(dipropylammonio)propyl]azepanium dichloride (**1o**), respectively, was only slightly lower compared to activity of the substances **1p** and **1h** (Table 6 and Table 7).

It was found that *MT*_v_ H_37_R_v_ was not so sensitive to the compounds **1a**–**p** as to INH (*MIC* = 0.5 μM and 1 μM). The most promising substances **1d**, **1h**, **1l**, **1n** or **1p** showed *MIC* = 4 μM and 8 μM (Table 6), respectively.

Similar trends were observed when evaluating susceptibility of *MK* 235/80. The molecules **1h**, **1n** and **1p** showed *MIC* = 8 μM, however, the OFLX reference drug was more active (*MIC* = 0.5 μM and 1 μM; Table 6). Comparable ability to fight *MK* 6509/96 was found for the derivative **1h**, series **1n**–**p** (*MIC* = 4 μM and 8 μM) and INH (*MIC* = 2–8 μM; Table 6), respectively.

All 3-alkoxy substituted molecules **1e**–**h** and **1m**–**p** were more active against *MT*_a_ H_37_R_a_ (*MIC* = 3.7–8.1 μM) than INH (*MIC* = 36.5 μM) or CPX (*MIC* = 48.3 μM). The dibasic compounds were also able to fight *MK* DSM more effectively, their *MIC* values ranged from 1.9 μM (**1h**) to 16.2 μM (**1e**), than INH (*MIC* = 29.2 μM; Table 6). Moreover, the derivatives **1g**, **1h** and **1n**–**p** were almost equally efficient against *MA* 330/80 as EMB (*MIC* = 16 μM; Table 7).

If attention was paid to the 2-alkoxy substituted compounds **1a**–**d** and **1i**–**l**, 1-[2-[({[2-(heptyl-oxy)phenyl]amino}carbonyl)oxy]-3-(dipropylammonio)propyl]pyrrolidinium dichloride (**1d**) was more active against both *MT*_a_ H_37_R_a_ (*MIC* = 12.5 μM) and *MK* DSM (*MIC* = 6.2 μM) than INH (Table 6).

**Figure 5:**
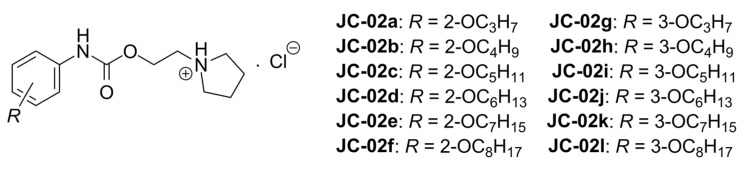
Chemical structure of the derivatives of 2-/3-alkoxyphenylcarbamic acids **JC-02a**–**l** (alkoxy = propoxy to octyloxy), which were *in vitro* screened against *MT*_v_ H_37_R_v_, *MK* 235/80, *MK* 6509/96 and *MA* 330/80 [76], respectively.

Waisser et al. [76] *in vitro* tested two series of structurally similar molecules **JC-01a**–**l** (Figure 3) and **JC-02a**–**l** (Figure 5) against *MT*_v_ H_37_R_v_, *MK* 235/80, *MK* 6509/96 and *MA* 330/80, respectively. The authors found that the compounds with an azepanium group were antimycobacterially more promising agents than the ones containing a pyrrolidinium heterocycle.

Current *in vitro* assays revealed that both branching of a connecting hydrocarbon chain and presence of ‘an additional’ dipropylammonium group within a salt-forming part of tested compounds **1a**–**p** were favorable for their antimycobacterial activity and they were more effective than the sets **JC-01a**–**l** and **JC-02a**–**l**.

The INH standard was less effective against *MS* (*MIC* = 117 μM) as well as *MM* (*MIC* = 467 μM) than almost all screened phenylcarbamic acid-based substances, exluding the derivatives **1a**, **1i**, **1k** or **1l** (Table 7). 1-[2-[({[2-(Pentyloxy)phenyl]amino}carbonyl)oxy]-3-(dipropylammonio)propyl]-azepanium oxalate (**1j**) was the most active against *MS* (*MIC* = 6.2 μM), the molecule **1h** fight *MM* most efficiently (*MIC* < 3.7 μM). On the other hand, these strains were sensitive to lower MICs of CPX (*MIC* = 0.4 μM and 0.8 μM; Table 7). It should be also mentioned that both sets **JC-01a**–**l** (Figure 3) and **JC-02a**–**l** (Figure 5) have not been *in vitro* tested against *MT*_a_ H_37_R_a_, *MK* DSM, *MS* or *MM*.

The chemometric PCA tool was applied to study activity–activity relationships of the compounds **1a**–**p** to explore similarities or differences in their impact on particular mycobacterial strains. The analysis was carried out by the XLSTAT *ver.* 2016.02.28451 software (Addinsoft, New York, NY, USA), a statistical application for Microsoft Excel *ver.* 2013 (Microsoft Corp., Redmont, WA, USA). Number of PCs was determined using visual evaluation of a scree plot, as a relationship between calculated *λ*_e_ and number of PCs. A present selection of PCs was based on a Kaiser–Guttman rule, as the most common stopping rule in PCA [77], which aimed an average value of *λ*_e_ > 1.0. The first two interpreted PCs of the analysis (with *λ*_e_ > 1.0) accounted for 89.59% of the total variance in the data as follows: 77.22% (PC 1) and 12.37% (PC 2), respectively.

**Figure 6:**
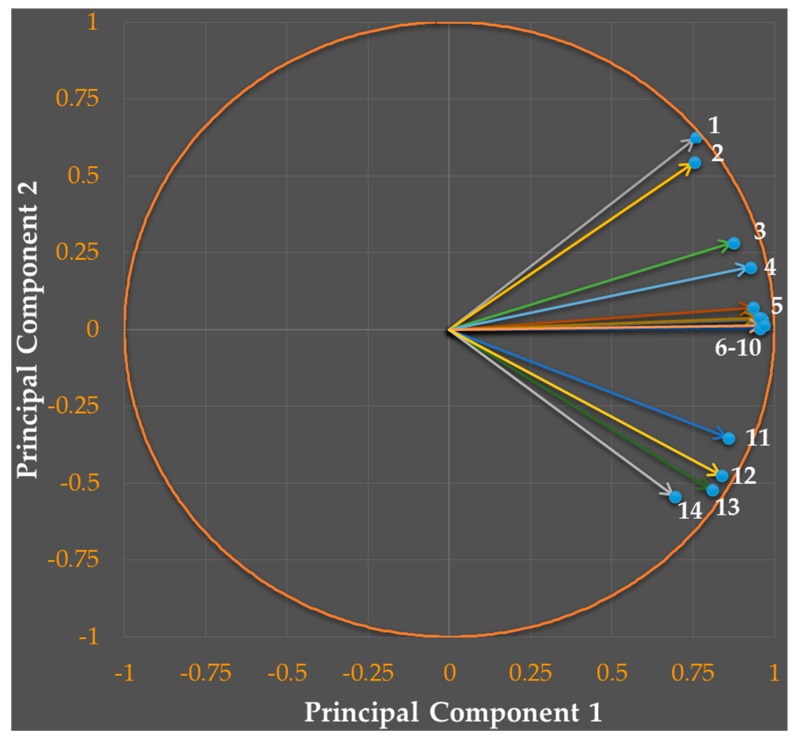
Two-dimensional mapping of the loadings of variables (variously colored vectors) indicating their (i) positions towards a circle of correlation; and (ii) relationships to both Principal Component 1 and 2. Numbering of the vectors was as follows: 1 (the vector built on the log (1/*MIC* [M]) values, which were observed after 14-d *in vitro* cultivation against *MT*_v_ H_37_R_v_), 2 (*MT*_v_ H_37_R_v_, 21-d), 3 (*MK* 235/80, 7-d), 4 (*MA* 330/80, 14-d), 5 (*MK* 235/80, 21-d), 6 (*MK* 235/80, 14-d), 7 (*MK* 6509/96, 14-d), 8 (*MK* 6509/96, 7-d), 9 (*MA* 330/80, 21-d), 10 (*MK* 6509/96, 21-d), 11 (*MT*_a_ H_37_R_a_, 7-d), 12 (*MM*, 21-d), 13 (*MK* DSM, 7-d) and 14 (*MS*, 3-d), respectively.

The quality of 2D representation of a variable was visualized by a distance between the projected variable onto a plane and circle of correlation. The loadings of variables were indicated as variously colored vectors, connected with particular mycobacterial strains, and numbered according to their position in the circle of radius 1 in an absolute value (Figure 6). In more detail, the digit 1 was assigned to the vector built on the log (1/*MIC* [M]) values, which were connected with 14-d *in vitro* testing of the compounds **1a**–**p** against *MT*_v_ H_37_R_v_ (*MT*_v_ H_37_R_v_, 14-d). Analogously, numbering of other vectors was as follows: 2 (*MT*_v_ H_37_R_v_, 21-d), 3 (*MK* 235/80, 7-d), 4 (*MA* 330/80, 14-d), 5 (*MK* 235/80, 21-d), 6 (*MK* 235/80, 14-d), 7 (*MK* 6509/96, 14-d), 8 (*MK* 6509/96, 7-d), 9 (*MA* 330/80, 21-d), 10 (*MK* 6509/96, 21-d), 11 (*MT*_a_ H_37_R_a_, 7-d), 12 (*MM*, 21-d), 13 (*MK* DSM, 7-d) and 14 (*MS*, 3-d), respectively.

A position of the variable in mapping could be defined by values of square cosines (cos2), which estimated quality of representation. The sum of cos2 for variables on PCs is equal to one. The higher cos2, the more perfect representation of a variable by chosen PCs [68]. The highest sum of cos2 on PC 1 and PC 2 was found for the vector 1 (*MT*_v_ H_37_R_v_, 14-d). This vector was characterized by cos2 = 0.961 (Table S8) indicating its almost exclusive representation by both PC 1 and PC 2. In addition, its position was the closest to a circle of correlation (Figure 6) compared to positions of the vectors 2–14. The cos2 values on particular PCs related to concerned vectors are listed in Table S8.

The vectors 1–3 were defined by PC 1 > 0.75 and PC 2 > 0.25, the vectors 4–10 were described by PC 1 > 0.75 and PC 2, which could be found in an interval from 0.25 to -0.25. Furthermore, PC 1 > 0.75 together with PC 2 between −0.35 and −0.53 were used to characterize the vectors 11–13. Finally, the vector 14 was described by PC 1 < 0.75 and PC 2 < −0.50 (Figure 6), respectively.

Visual assessment indicated the smallest angles between the vectors 5–10. A relatively sharp angle was also observed between 1 and 2 as well as 12 and 13, respectively. Conversely, there was found ‘almost orthogonal’ arrangement of the vectors 1 and 14. Regarding this visualization, it could be assumed that the compounds **1a**–**p** showed (i) similar mechanisms of action against *MK* 235/80, *MK* 6509/96 and *MA* 330/80, respectively; (ii) different mechanisms of action against *MT*_v_ H_37_R_v_ and *MS*.

### 2.4. Structure–Activity Relationships

As suggested in previous sections of the paper, surface tension (relative surface activity γ; in N/m units), electronic (log *ε*_2 (Ch-T)_) and lipophilic (log *k*_w_) properties of the compounds **1a**–**p** might notably affect their *in vitro* efficiency (in log (1/*MIC* [M]) units) against tested mycobacterial strains. The PCA approach was applied for preliminary *SAR* studies of these derivatives by the XLSTAT *ver.* 2016.02.28451 software. Number of PCs was determined using visual evaluation of a scree plot, as a relationship between calculated *λ*_e_ and number of PCs. The first three PCs of the analysis with *λ*_e_ > 1.0 [77] accounted for 90.01% of the total variance in the data as follows: 69.59% (PC 1), 14.35% (PC 2) and 6.07% (PC 3), respectively.

The 2D visualization of relationships between the vectors describing physicochemical parameters and *in vitro* antimycobacterial activities of the compounds **1a**–**p** on axes (i) PC 1 and PC 2 (Figure S2), as well as (ii) PC 1 and PC 3 (Figure S3) revealed the closest connection between lipophilicity (log *k*_w_; vector A) and efficiency against *MT*_v_ H_37_R_v_ (14-d; 1). In addition, there were found notably negatively correlated variables (vectors), which were positioned on opposed quadrants of the loading plots (Figures S2 and S3). These correlations aimed γ (C) versus log *k*_w_ (A) or C versus the vectors built on *in vitro* activity against *MT*_v_ H_37_R_v_ (14-d; 1), *MT*_v_ H_37_R_v_ (21-d; 2), *MA* 330/80 (14-d; 3) as well as *MK* 235/80 (7-d; 4). It seemed that ‘almost orthogonal’ arrangement of the vector characterizing log *ε*_2 (Ch-T)_ (B) to those of antimycobacterial activities (5–14; Figure S3) indicated no correlation.

A linear, quasi-parabolic (polynomial function of 2^nd^ order) and sigmoidal function, respectively, was also employed in order to investigate *SAR* in more detailed manner. The research focused on relationships between the independent variables (γ, log *ε*_2 (Ch-T)_, log *k*_w_) and dependent variable(s), i.e., biological values (in log (1/*MIC* [M]) units).

Resulting equations and values of common statistical descriptors, namely, *n*, *DF*, *χ*,^2^_red_, *RSS*, *R*, *Adj. R*^2^, *RMSE*, *NR*, *F* and *Prob* > *F*, respectively, were calculated by using the Origin Pro *ver.* 9.0.0 SR2 software. In addition, only statistically significant (*Prob > F* values varied from 0.0100 to <0.0500; one-star indication), very significant (*Prob > F* from 0.0010 to <0.0100; two stars indication) or extremely significant (*Prob > F* from 0.0000 to <0.0010; three stars indication) relationships between the independent and dependent variables were presented in next paragraphs. These models were also characterized by *R* ≥ 0.9000, *Adj. R*^2^ ≥ 0.7750 and *F* ≥ 25.00, respectively.

Respecting given criteria, the following discussion considered relationships connected with *MT*_v_ H_37_R_v_, *MK* 235/80, *MK* 6509/96, *MK* DSM, *MA* 330/80 and *MS* as well. Conversely, there were found no statistically significant, very significant neither extremely significant models related to *MT*_a_ H_37_R_a_ or *MM*. It should be also noted that suggested models meeting those requirements were connected mostly with the subgroups containing a limited number of compounds (*n* = 8). However, it was not possible to perform the specific PCA for such subsets due to this restriction, i.e., number of effective observations was lower (*n* = 8) than number of examined variables (*n* = 17). This was a reason why next sections were focused mainly on linear, quasi-parabolic or sigmoidal fitting procedures and their statistical descriptions.

#### 2.4.1. Models Related to *Mycobacterium tuberculosis* CNCTC My 331/88

Notable linear relationships between the γ or log *k*_w_ parameters and log (1/*MIC* [M]) values based on both 14-d and 21-d *in vitro* screenings against *MT*_v_ H_37_R_v_ were observed for the series **1a**–**h**. These models were defined by Equations (3)–(6) and found to be statistically very significant or extremely significant. Decrease in γ and increase in log *k*_w_ of these derivatives led to their more promising antitubercular profile.

*MT*_v_ H_37_R_v_ (14-d)/**1a**–**h**: log (1/*MIC* [M]) = –120.2060 (±12.9578) × γ + 12.3509 (±0.8027) (3)

*n* = 8, *DF* = 6, *χ*,^2^_red_ = 0.0058, *RSS* = 0.0347, *R* = 0.9669, *Adj. R*^2^ = 0.9240, *RMSE* = 0.0761, *NR* = 0.1863, *F* = 86.06, *Prob > F* = 0.0001 ***

*MT*_v_ H_37_R_v_ (14-d)/**1a**–**h**: log (1/*MIC* [M]) = 0.4301 (±0.0737) × log *k*_w_ + 2.9438 (±0.3393) (4)

*n* = 8, *DF* = 6, *χ*,^2^_red_ = 0.0133, *RSS* = 0.0798, *R* = 0.9220, *Adj. R*^2^ = 0.8251, *RMSE* = 0.1153, *NR* = 0.2825, *F* = 34.03, *Prob > F* = 0.0011 **

*MT*_v_ H_37_R_v_ (21-d)/**1a**–**h**: log (1/*MIC* [M]) = –103.4692 (±18.7975) × γ + 11.1642 (±1.1644) (5)

*n* = 8, *DF* = 6, *χ*,^2^_red_ = 0.0122, *RSS* = 0.0730, *R* = 0.9136, *Adj. R*^2^ = 0.8072, *RMSE* = 0.1103, *NR* = 0.2702, *F* = 30.30, *Prob > F* = 0.0015 **

*MT*_v_ H_37_R_v_ (21-d)/**1a**–**h**: log (1/*MIC* [M]) = 0.3957 (±0.0633) × log *k*_w_ + 2.9505 (±0.2911) (6)

*n* = 8, *DF* = 6, *χ*,^2^_red_ = 0.0098, *RSS* = 0.0587, *R* = 0.9312, *Adj. R*^2^ = 0.8449, *RMSE* = 0.0989, *NR* = 0.2424, *F* = 39.13, *Prob > F* = 0.0008 ***

It seemed that ability to decrease surface tension of water was slightly more important than increase in lipophilicity of inspected compounds **1a**–**h** with the intention to improve their antitubercular properties, especially when concerning 14-d *in vitro* biological evaluation.

It would not be reasonable to suggest some biparametric linear models containing γ, log *k*_w_ and log (1/*MIC* [M]) parameters with an attempt to provide more satisfactory values of statistical descriptors related to the set **1a**–**h**. The reason not to apply bilinear reggresion analyses would be that statistically extremely significant relationships between γ and log *k*_w_ (Equations (S25)–(S28) in Table S7) were already observed.

Current findings were only in partial agreement with a research of Čižmárik et al. [38,78,79], who studied derivatives of 2-/3-alkoxyphenylcarbamic acid (alkoxy = propoxy do decyloxy), which basic part was formed by only one azacycloalkyl moiety (pyrrolidinium or piperidinium). The authors employed a linear, quasi-parabolic and sigmoidal model, respectively, to provide comprehensive *QSAR* investigation. They found that simultaneous increase in lipophilicity and *in vitro* antitubercular activity of these molecules was quite limited [78,79]. In addition, this behavior was also observed when inspecting relationships between the *in silico* log *p* values (CLOGP 4.0) and *in vitro* efficiency of these monobasic compounds against non-tuberculous *MK* 235/80, *MK* 6509/96 and *MA* 330/80, respectively [38].

The antitubercular activities of derivatives **1a**–**p** might theoretically indicate that comparable efficiency of homologues containing a pyrrolidinium group would be observed, if length of their side chain *R* would be beyond a certain border, i.e., if *R* = 2-/3-OC_8_H_17_ or 2-/3-OC_9_H_19_.

A cell envelope of *MT*_v_ H_37_R_v_ is composed of three major segments, namely, a plasma membrane, cell wall core and outermost layer. The cell wall core, which is essential for viability, consists of peptidoglycan (PG) in covalent attachment *via* phosphoryl-*N*-acetylglucosaminosyl-rhamnosyl linkage units with heteropolysaccharide arabinogalactan (AG). The AG is in turn esterified at its non-reducing ends to long-chain (C_70_–C_90_) mycolic acids. The latter form the bulk of the inner leaflet of the outer membrane, with the outer layer consisting of a variety of non-covalently attached (glyco)lipids, polysaccharides, lipoglycans, and proteins [80].

Results of present statistical analyses indicated that not only lipophilic nature of the compounds **1a**–**p**, possible interactions of their side chain *R* with some components of the cell wall or ability do decrease surface tension of water in order to ‘non-specifically’ disrupt the cell envelope could be decisive for their *in vitro* efficiency. In fact, suggested mechanisms of antitubercular action might become more relevant for the subgroup **1a**–**h** compared to **1i**–**p**.

A closer look at the relationships between inspected physichochemical and biological descriptors among particular positional isomers proved that there were calculated more convenient statistical values for the 2-alkoxy substituted compounds **1a**–**d** and **1i**–**l** (Equations (S29)–(S32) in Table S9) compared to those of the 3-alkoxy substituted isomers **1e**–**h** and **1m**–**p**, respectively.

#### 2.4.2. Models Related to the *Mycobacterium kansasii* Species

Statistically significant linear relationships between the log *k*_w_ parameters and log (1/*MIC* [M]) values connected with *in vitro* testing of the series **1a**–**h** against *MK* 235/80 were found. The *MIC* values observed after 7-d and 14-d cultivation were the same (Table 6), so both proposed models were defined by Equation (7). More prospective anti-*MK* 235/80 compounds showed higher log *k*_w_ values.

*MK* 235/80 (14-d)/**1a**–**h**: log (1/*MIC* [M]) = 0.4395 (±0.0650) × log *k*_w_ + 2.6388 (±0.2990) (7)

*n* = 8, *DF* = 6, *χ*,^2^_red_ = 0.0103, *RSS* = 0.0620, *R* = 0.9403, *Adj. R*^2^ = 0.8648, *RMSE* = 0.1016, *NR* = 0.2489, *F* = 45.76, *Prob > F* = 0.0001 ***

Statistically extremely significant linear models between the log *k*_w_ and log (1/*MIC* [M]) values resulting from 7-d and 21-d *in vitro* screening of the subgroup **1a**–**h** against *MK* 6509/96 were also set. The observed dependences were defined by Equations (8) and (9). Higher log *k*_w_ values were related to more promising anti-*MK* 6509/96 agents.

*MK* 6509/96 (7-d)/**1a**–**h**: log (1/*MIC* [M]) = 0.5072 (±0.0757) × log *k*_w_ + 2.5914 (±0.3485) (8)

*n* = 8, *DF* = 6, *χ*,^2^_red_ = 0.0140, *RSS* = 0.0842, *R* = 0.9392, *Adj. R*^2^ = 0.8624, *RMSE* = 0.1185, *NR* = 0.2901, *F* = 44.86, *Prob > F* = 0.0001 ***

*MK* 6509/96 (21-d)/**1a**–**h**: log (1/*MIC* [M]) = 0.5061 (±0.0737) × log *k*_w_ + 2.3356 (±0.3393) (9)

*n* = 8, *DF* = 6, *χ*,^2^_red_ = 0.0133, *RSS* = 0.0798, *R* = 0.9418, *Adj. R*^2^ = 0.8682, *RMSE* = 0.1153, *NR* = 0.2825, *F* = 47.12, *Prob > F* = 0.0005 ***

Increase in lipophilicity of the derivatives **1a**–**h** was more important factor to positively influence their efficiency compared to surface properties. However, it could be assumed that constant increase in lipophilicity due to elongation of the side chain *R* would not led to more promising derivatives, as observed for a subgroup **1i**–**p** (Table 6).

Statistically very significant and extremely significant linear relationships between the γ or log *k*_w_ and log (1/*MIC* [M]) descriptors were also found when concerning the molecules **1a**–**h** and their 7-d *in vitro* efficiency against *MK* DSM. The models were characterized by Equations (10) and (11), respectively.

Highly lipophilic compounds with ability to decrease surface tension of water more markedly showed higher potential to fight given *mycobacterium*. Conversely, increase in lipophilicity due to increase in number of carbons of the azacycloalkyl fragment (**1i**–**p**) would not be a guaranty of improvement in anti-*MK* DSM activity (Table 6).

*MK* DSM (7-d)/**1a**–**h**: log (1/*MIC* [M]) = –192.0357 (±33.5781) × γ + 16.8889 (±2.0800) (10)

*n* = 8, *DF* = 6, *χ*,^2^_red_ = 0.0388, *RSS* = 0.2330, *R* = 0.9192, *Adj. R*^2^ = 0.8192, *RMSE* = 0.1971, *NR* = 0.4827, *F* = 32.71, *Prob > F* = 0.0012 **

*MK* DSM (7-d)/**1a**–**h**: log (1/*MIC* [M]) = 0.7579 (±0.0817) × log *k*_w_ + 1.5371 (±0.3758) (11)

*n* = 8, *DF* = 6, *χ*,^2^_red_ = 0.0163, *RSS* = 0.0979, *R* = 0.9670, *Adj. R*^2^ = 0.9240, *RMSE* = 0.1277, *NR* = 0.3129, *F* = 86.15, *Prob > F* = 0.0001 ***

#### 2.4.3. Models Related to *Mycobacterium avium* CNCTC My 330/80

Different models were suggested when exploring relationships between γ and log (1/*MIC* [M]) values resulting from 14-d and 21-d *in vitro* screening of the compounds **1a**–**p** against *MA* 330/80. Concerning well-known differences in their salt-forming fragment, a statistically very significant linear model was found for a subgroup **1a**–**h** (14-d) and defined by Equation (12).

*MA* 330/80 (14-d)/**1a**–**h**: log (1/*MIC* [M]) = –150.5250 (±29.7405) × γ + 13.7803 (±1.8423) (12)

*n* = 8, *DF* = 6, *χ*,^2^_red_ = 0.0305, *RSS* = 0.1828, *R* = 0.9001, *Adj. R*^2^ = 0.7790, *RMSE* = 0.1745, *NR* = 0.4275, *F* = 25.61, *Prob > F* = 0.0032 **

**Figure 7:**
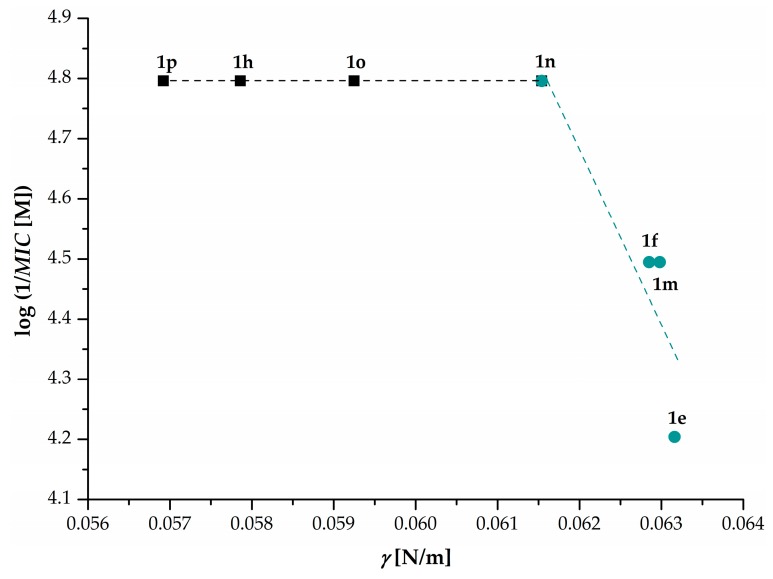
Bilinear relationship between the γ (in N/m units) and log (1/*MIC* [M]) parameters resulting from 21-d *in vitro* screening of the 3-alkoxy substituted compounds **1e**–**h** and **1m**–**p** (alkoxy = butoxy to heptyloxy) against *MA* 330/80.

If a main criterion to consider was a position of compounds’ side chain *R*, a bilinear model between γ and log (1/*MIC* [M]) was regarded as the most convenient for two subsets **1e**–**h** and **1m**–**p** (Figure 7).

A bilinear model proposed by Kubinyi [81] was generally applicable and presented a smooth synthesis of both linear and non-linear parts of *SAR* as an effort to simulate a complex process in a rather simplistic way.

The compounds with γ varying from 0.05692 N/m (**1p**) to 0.06154 N/m (**1n**) showed a comparable capability to act against *MA* 330/80 (21-d). Further increase in γ, i.e., minor ability to decrease surface tension of water, resulted in sharp decrease in their antimycobacterial efficiency. The observed behavior could indicate a non-specific mechanism of action of these derivatives, which might be based on their ability to disrupt an outermost layer of a cell envelope of *MA* 330/80. The layer, which consists of diverse amphiphilic glycolipids, namely, mycosides C, glycolipids, peptidolipids, and phospholipids, respectively, hinders diffusion of chemotherapeutic agents *via* the wall thus causing multiple drug-resistance by exclusion [82].

Statistically very significant or extremely significant linear relationships between log *k*_w_ and log (1/*MIC* [M]) resulting from 14-d and 21-d *in vitro* screening of the compounds **1a**–**h** were also observed. The suggested models were defined by Equations (13) and (14). As expected, higher log *k*_w_ values were connected with more effective agents.

*MA* 330/80 (14-d)/**1a**–**h**: log (1/*MIC* [M]) = 1.6968 (±0.3122) × log *k*_w_ + 0.6051 (±0.0678) (13)

*n* = 8, *DF* = 6, *χ*,^2^_red_ = 0.0113, *RSS* = 0.0675, *R* = 0.9643, *Adj. R*^2^ = 0.9182, *RMSE* = 0.1061, *NR* = 0.2599, *F* = 79.57, *Prob > F* = 0.0001 ***

*MA* 330/80 (21-d)/**1a**–**h**: log (1/*MIC* [M]) = 1.8560 (±0.4695) × log *k*_w_ + 0.5458 (±0.1020) (14)

*n* = 8, *DF* = 6, *χ*,^2^_red_ = 0.0255, *RSS* = 0.1528, *R* = 0.9092, *Adj. R*^2^ = 0.7978, *RMSE* = 0.1596, *NR* = 0.3909, *F* = 28.62, *Prob > F* = 0.0081 **

Statistical characteristics given above might support a hypothesis, which was based on a non-specific mechanism of action of the molecules **1a**–**h** against *MA* 330/80. However, the model related to 21-d *in vitro* biological evaluation (Equation (14)) provided clearly worse values of the statistical descriptors than those connected with 14-d *in vitro* screening (Equation (13)).

**Figure 8:**
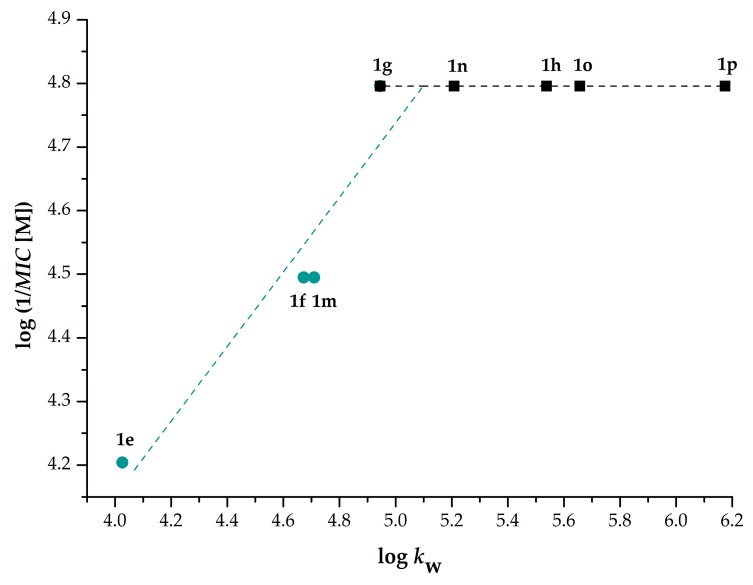
Bilinear relationship between the log *k*_w_ and log (1/*MIC* [M]) parameters resulting from 21-d *in vitro* screening of the 3-alkoxy substituted compounds **1e**–**h** and **1m**–**p** (alkoxy = butoxy to heptyloxy) against *MA* 330/80.

If attention was turned to the 3-alkoxy substituted derivatives **1e**–**h** and **1m**–**p**, increase in their log *k*_w_ up to approximately 5.1000 (Table 3) meant sharp increase in activity against *MA* 330/80 (21-d). Further increase in lipophilicity beyond this border was not reflected in higher antimycobacterial potential of the compounds **1n**, **1h**, **1o** and **1p** showing log *k*_w_ of 5.2087, 5.5384, 5.6569 and 6.1749 (Table 3, Figure 8), respectively.

#### 2.4.4. Models Related to *Mycobacterium smegmatis* ATCC 700084

There was observed a very significant quasi-parabolic relationship between the log *ε*_2 (Ch-T)_ values and log (1/*MIC* [M]) parameters connected with 3-d *in vitro* evaluation of the pyrrolidium moiety-containing compounds **1c**–**h** against *MS* (Equation (S33) in Table S9). The possibility to exclude the molecules **1a** and **1b** from a current analysis was based on the observation that they showed the lowest efficiency against given *mycobacterium* (Table 7).

It was found that anti-*MS* activity increased with increasing log *ε*_2 (Ch-T)_, reached a maximum if this independent variable was between 4.24 and 4.27 (**1e**, **1f** and **1h**) and then decreased with further increase in the log *ε*_2 (Ch-T)_ values (Figure S4, Table 1). This behavior, defined as a cut-off effect, was originally reviewed and rationalized in number of mechanistic ways by Hansch and Clayton a few decades ago [83].

It was also found that lower γ and higher log *k*_w_ did not lead to increase in anti-*MS* activity of the compounds **1i**–**p**, as explored by some models, which were not provided because of their statistical insignificance (*Prob* > *F* ≥ 0.0500).

## 3. Materials and Methods

### 3.1. General Information

Liquid chromatography high resolution mass spectroscopy (HPLC-HR-MS) analyses of the compounds **9′a**–**p** (Supplementary Materials) were performed on a chromatographic apparatus consisting of the LC Agilent Infinity System (Agilent Technologies, Santa Clara, CA, USA) equipped with a gradient pump (1290 Bin Pump VL), automatic injector (1260 HiPals), and column thermostat (1290 TCC). The LC system was coupled with the Quadrupole Time-Of-Flight mass spectrometer (6520 Accurate Mass Q-TOF LC/MS). Q-TOF was equipped with an electrospray ionization source operated in a positive and negative ionization mode as well.

For data acquisition and processing, a personal computer with the Mass Hunter software *ver*. MassHunter Workstation B 04.00 (Agilent Technologies) was used.

Each of the compounds **9′a**–**p** was dissolved in 50% (*v*/*v*) MeOH to reach a concentration of 1 mg/L. Particular solutions were filtered via 0.22 µm nylon syringe filter and 1.0 µL was used for the HPLC-HR-MS analyses at 35 °C using the RP-C_18_ column Zorbax Extend-C_18_, 2.1 × 50 mm, 1.7 µm (Agilent Technologies).

The mobile phases consisted of 0.1% aqueous solution of formic acid in demineralized water (mobile phase A) and acetonitrile (mobile phase B). Gradient elution was used with linear gradient from 5% to 95% of acetonitrile *per* 8 min. The flow rate of a mobile phase was set to 400 µL/min.

The MS spectrometer was operated in a positive and negative ionization mode, respectively, keeping particular specifications as follows: drying gas temperature 360 °C, drying gas flow 12 L/min, nebulizing gas pressure 60 psi, ESI source voltage 4000 V, fragmentor voltage 125 V, skimmer voltage 65 V and collision gas N_2_, respectively. The *m*/*z* ratios of both [M + H]^+^ and [M − H]^−^ ions were recorded in an interval from 50 *m*/*z* to 1500 *m*/*z*. Observed *m*/*z* values of the adducts were compared to the theoretical ones. The difference (in ppm units; Supplementary Materials) was calculated according to Equation (15):

*Difference =* [(*m*/*z*_theoretical_ − *m*/*z*_obtained_)/*m*/*z*_theoretical_] × 10^6^ (15)

High-resolution mass spectra of the compounds **1a**–**p** were measured using the Dionex UltiMate 3000 high-performance liquid chromatograph (Thermo Scientific, West Palm Beach, FL, USA) coupled with the LTQ Orbitrap XL Hybrid Ion Trap-Orbitrap Fourier Transform Mass Spectrometer (Thermo Scientific) equipped with a HESI II (heated electrospray ionization) source operating in a positive (**1a**–**d**, **1i**–**l**) or negative (**1e**–**h**, **1m**–**p**) mode. The HPLC system was controlled through the Chromeleon Chromatography Data System *ver*. 7.2 (Thermo Scientific). The separation was performed on a C_18_-Hypersil Gold (3 µm, 50 mm × 2.1 mm) column (Thermo Scientific). A mobile phase consisted of water Purelab Classic (ELGA LabWater, High Wycombe, Bucks, UK) and acetonitrile hypergrade for LC-MS LiChrosolv (Merck KGaA, Darmstadt, Germany) in 80:20 volume ratio (*v*/*v*). Total flow rate was 0.3 mL/min, injection volume was 1 μL, and column temperature was set to 30 °C, respectively.

The UV/Vis spectra of methanolic solutions of analyzed compounds **1a**–**p** (*c* = 8.0 × 10^−5^ M) were observed on the 8452A Diode Array spectrophotometer HP-8452A (Hewlett-Packard, Palo Alto, CA, USA) at 21 °C. Methanol for UV-spectroscopy (Merck, Darmstadt, Germany) was used for preparation of these solutions. Results of the UV/Vis analyses were collected and stored digitally using the ChemStation controller software (Agilent Technologies, Waldbronn, Germany). The HP-8452A apparatus measured a complete range of compounds’ spectrum from 190 nm to 820 nm.

The HPLC separation module Waters e2695 equipped with a Waters 2996 PDA Detector (Waters Corp., Milford, MA, USA) and chromatographic column Symmetry C_18_ × 5 μm, 4.6 × 250 mm, Part No. W21751W016 (Waters Corp.) were used for estimation of lipophilic properties of the compounds **1a**–**p** at 21 °C.

The HPLC separation process was monitored by the Empower 3 Chromatography Data Software (Waters Corp.) working on a personal computer HP Compaq (Hewlett-Packard, Palo Alto, CA, USA) equipped with Intel^®^ Core^TM^ i5 processor (Intel Corp., Santa Clara, CA, USA), 2400 CPU, 3.10 GHz, and 4.00 GB of RAM, respectively. The computer operated on the Windows 7 Professional system, a 64-bit version (Microsoft Corp., Redmont, WA, USA).

Isocratic elution was carried out using mobile phases consisting of methanol *p.a.* (MeOH; Honeywell, Paris, France) and purified water in various volume ratios (80:20, 85:15, 90:10, 95:5 (*v*/*v*) and pure MeOH, respectively). Distilled water (CentralChem, Bratislava, Slovak Republic) was purified by the Aquinity2 P10 ultra-pure water system for production of ultra-pure water with an integrated 10 L permeate tank (membraPure, Hennigsdorf, Germany).

Total flow of the column was 1.0 mL/min, injection volume was 4 μL and column temperature was set to 40 °C. The detection wavelengths varying from 236 nm to 238 nm were chosen for current experimental procedures.

### 3.2. Synthesis of Compounds

Presently analyzed 1-[2-[({[2-/3-(alkoxy)phenyl]amino}carbonyl)oxy]-3-(dipropylammonio)- propyl]pyrrolidinium oxalates (**1a**–**d**)/dichlorides (**1e**–**h**) as well as 1-[2-[({[2-/3-(alkoxy)phenyl]- amino}carbonyl)oxy]-3-(dipropylammonio)propyl]azepanium oxalates (**1i**–**l**)/dichlorides (**1m**–**p**; alkoxy = butoxy to heptyloxy) were prepared by multi step pathways (Scheme 1) using 2-aminophenol (**1a**) and 3-aminophenol (**1b**), respectively, as starting compounds [21,27,28,29].

Procedures for preparation of reaction intermediates **2′a**, **2′b**, **3′a**–**h**, **4′a**–**h**, **5′a**–**h**, **7′**, **8′a**, **8′b**, **9′a**–**p** and final molecules **1a**–**p** were originally published in research papers [21,27,28,29] and are provided in Supplementary Materials. In addition, HPLC-HR-MS spectral characterizations of the compounds **9′a**–**p** are included as well.

Spectral data (IR), elemental analyses results (% C, H, N), melting point (m.p.) values, *R*_f_ parameters (TLC) and acid-base dissociation constants (p*K*_a1_, p*K*_a2_), respectively, of solid colorless compounds **1a**–**p** can be found in the papers [21,26] and some of given characteristics are also listed in Supplementary Materials.

The molecules **1a**–**p** were re-crystallized from mixture of acetone/ethanol (**1a**–**d, 1e**–**h, 1m**–**p**) or acetone (**1i**–**l**) before being spectrally, physicochemically and biologically investigated. Present HR-MS spectral description of these compounds was given below.

The compound **1a**: HR-MS for C_24_H_42_N_3_O_3_ [M + H]^+^ calculated 420.32207 *m*/*z*, found 420.32343 *m*/*z*; **1b**: HR-MS for C_25_H_44_N_3_O_3_ [M + H]^+^ calculated 434.33772 *m*/*z*, found 434.33920 *m*/*z*; **1c**: HR-MS for C_25_H_46_N_3_O_3_ [M + H]^+^ calculated 448.35337 *m*/*z*, found 448.35471 *m*/*z*. **1d**: HR-MS for C_27_H_47_N_3_O_3_ [M + H]^+^ calculated 462.36902 *m*/*z*, found 462.37048 *m*/*z*; **1e**: HR-MS for C_24_H_40_N_3_O_3_ [M − H]^−^ calculated 418.30751 *m*/*z*, found 418.30746 *m*/*z*; **1f**: HR-MS for C_25_H_42_N_3_O_3_ [M − H]^−^ calculated 432.32316 *m*/*z*, found 432.32294 *m*/*z*; **1g**: HR-MS for C_26_H_44_N_3_O_3_ [M − H]^−^ calculated 446.33881 *m*/*z*, found 446.33912 *m*/*z*; **1h**: HR-MS for C_27_H_46_N_3_O_3_ [M − H]^−^ calculated 460.35446 *m*/*z*, found 460.35458 *m*/*z*; **1i**: HR-MS for C_26_H_46_N_3_O_3_ [M + H]^+^ calculated 448.35337 *m*/*z*, found 448.35458 *m*/*z*; **1j**: HR-MS for C_27_H_48_N_3_O_3_ [M + H]^+^ calculated 462.36902 *m*/*z*, found 462.37045 *m*/*z*; **1k**: HR-MS for C_28_H_50_N_3_O_3_ [M + H]^+^ calculated 476.38567 *m*/*z*, found 476.38608 *m*/*z*; **1l**: HR-MS for C_29_H_52_N_3_O_3_ [M + H]^+^ calculated 490.40192 *m*/*z*, found 490.40210 *m*/*z*; **1m**: HR-MS for C_26_H_44_N_3_O_3_ [M − H]^−^ calculated 446.33881 *m*/*z*, found 446.33908 *m*/*z*; **1n**: HR-MS for C_27_H_46_N_3_O_3_ [M − H]^−^ calculated 460.35446 *m*/*z*, found 460.35474 *m*/*z*; **1o**: HR-MS for C_28_H_48_N_3_O_3_ [M − H]^−^ calculated 474.37011 *m*/*z*, found 474.36996 *m*/*z*; **1p**: HR-MS for C_29_H_50_N_3_O_3_ [M − H]^−^ calculated 488.38576 *m*/*z*, found 488.38596 *m*/*z*.

### 3.3. Determination and Prediction of Physicochemical Properties

#### 3.3.1. Estimation of Surface Tension

Surface tension (relative surface activity; γ) of the derivatives **1a**–**p** was determined by a drop count method using a glassy Traube stalagmometer (Kavalier Glass, Prague, Czech Republic). Number of drops of compounds’ and reference solutions were counted between upper and lower marks of the stalagmometer [30,31]. Aqueous solutions of the molecules **1a**–**p** were prepared with distilled, deionized water (*c* = 2.0 × 10^−3^ M). Distilled deionized water was also used as a reference solution, which showed γ = 0.07259 N/m. The drops were allowed to form at constant temperature 21 °C. The experimental procedure was described in a paper [31]. Current measurements were done in six replicates and average γ values (in N/m units) were reported (Table 1).

#### 3.3.2. Estimation of Electronic Properties

The log *ε* values characterizing methanolic solutions of the compounds **1a**–**p** (*c* = 8.0 × 10^−5^ M) were observed at *λ*_1_ = 208–210 nm, *λ*_2 (Ch-T)_ = 236–238 nm and *λ*_3_ = 278–280 nm (Table 1), respectively, in a near ultraviolet (quarz) region of the electromagnetic spectrum between 200 nm and 400 nm [35]. The log *ε* values for presently observed absorption maxima were calculated following the Lambert-Beer’s law, which was discussed in [35], for example, and expressed by Equation (16):

*A* = *ε* × *c* × *l* (16)

where the *A* parameter represented absorbance of a compound’s solution, the *ε* descriptor was a molar absorption coefficient (in L/mol/cm units) and *l* was path length (in cm units).

#### 3.3.3. Estimation and *In Silico* Investigation of Lipohydrophilic Properties

Lipophilicity of the compounds **1a**–**p** was determined by reversed-phase high-performance liquid chromatography (RP-HPLC). Methanol (MeOH)/water mobile phases with varying volume ratio of the organic modifier and water (80:20, 85:15, 90:10, 95:5 (*v*/*v*) and pure MeOH, respectively) were chosen.

A methanolic solution of potassium iodine was used for dead time (*t*_d_) determination. Retention factors (capacity factors; *k*) were calculated according to Equation (17):

*k* = (*t*_r_*− t*_d_)/*t*_d_ (17)

where *t*_r_ was retention time of a solute (in min), the *t*_d_ parameter denoted dead time of potassium iodine, an unretained analyte (in min).

The observed retention (*t*_r_) and dead (*t*_d_) times were means of three independent determinations. Average *t*_d_ values of potassium iodine in used MPhs were as follows: 2.237 min (MeOH/water ratio (*v*/*v*) was 80:20), 2.245 min (85:15), 2.245 min (90:10), 2.228 min (95:5) and 2.210 min (pure MeOH), respectively.

The log *k*_w_ values, i.e., the logarithms of extrapolated retention (capacity) factors for 100% water in the isocratic RP-HPLC, were determined from intercepts of linear plots between the log *k* and *ϕ*_M_ (a volume fraction of an organic modifier in the isocratic elution RP-HPLC) according to Equation (18):

log *k* = log *k*_w_ − *S* × *ϕ*_M_ (18)

where the *S* parameter represented the slope of a regression curve, which was related to solvent strength of a pure organic solvent [51,52].

Purity (in percentages) of the compounds **1a**–**p** was also verified by RP-HPLC. Areas of their peaks were measured using the MPh, which contained 90% proportion (*v*/*v*) of MeOH. The purity of given salts is provided in Table S3.

The log *p* values of non-protonated bases **9a′**–**p** (Scheme 1, Table 4) for the octan-1-ol/water partitioning system were calculated by the Ghose and Crippen′s log *P*_Cr_ [54,55], Viswanadhan′s log *P*_V_ [56], Broto’s log *P*_B_ [57] and Leo’s CLOGP 4.0 [58] atomic as well as combined atomic and fragmental methods using the ChemBioDraw Ultra 12.0 software package (CambridgeSoft, Cambridge, MA, USA).

The Virtual Computer Chemistry Laboratory [59], a freely available web-based tool working in Java environment, was taken to generate log *P* by the Wang’s XLOGP 2.0 [60], Cheng’s XLOGP 3.0 [61], Moriguchi’s MLOGP [62], Sander’s (Actelion’s) ACLOGP [63], Molinspiration’s miLogP 2.2 [64], ALOGP [65] and Tetko’s ALOGPs 2.1 [66] method (Table 4 and Table 5), respectively. All these approaches integrated algorithms based on atomic/fragmental principles, excluding ALOGPs 2.1, which considered a molecule in the whole [66].

The SILICOS-IT hybrid method, which used a proprietary fragment- and property-based principles, was also employed to generate log *P* parameters (log *P*_S-IT_; Table 5). The method was implemented in the SwissADME applet, a free web tool designed to evaluate pharmacokinetics and drug-likeness of small molecules [67].

### 3.4. In Vitro Antimycobacterial Assays

The *in vitro* activity of compounds **1a**–**p** was inspected against *Mycobacterium tuberculosis* CNCTC My 331/88 (identical with H_37_R_v_ and ATCC 2794, respectively; abbreviation used: *MT*_v_ H_37_R_v_), *M. kansasii* CNCTC My 235/80 (identical with ATCC 12478; *MK* 235/80), the *M. kansasii* 6509/96 clinical isolate (*MK* 6509/96) and *M. avium* CNCTC My 330/80 (identical with ATCC 25291; *MA* 330/80), respectively, in the Laboratory for Mycobacterial Diagnosis and Tuberculosis (Institute of Public Health in Ostrava, Czech Republic). These strains were purchased from the National Reference Laboratory—Czech National Collection of Type Cultures (CNCTC; The National Institute of Public Health, Prague, Czech Republic), excluding *MK* 6509/96, which was clinically isolated because the INH-resistant *M. kansasii* strains have not been found in Czech Republic or Slovak Republic. In the experiments, dilution of the strains was as follows: 10^−3^ M (*MT*_v_ H_37_R_v_), 10^−4^ M (*MK* 235/80, *MK* 6509/96) and 10^−5^ M (*MA* 330/80), respectively.

The molecules **1a**–**p** were also *in vitro* screened against an avirulent *M. tuberculosis* H_37_R_a_ ATCC 25177 (*MT*_a_ H_37_R_a_), *M. kansasii* DSM 44162 (*MK* DSM), *M. smegmatis* ATCC 700084 (*MS*) and *M. marinum* CAMP 5644 (*MM*), respectively, in the Department of Infectious Diseases and Microbiology (Faculty of Veterinary Medicine, University of Veterinary and Pharmaceutical Sciences in Brno, Czech Republic).

*Standard drugs*. Isoniazid (INH), ethambutol (EMB), ofloxacin (OFLX) and ciprofloxacin (CPX) reference drugs were purchased from Sigma-Aldrich (Darmstadt, Germany), respectively, showing purity of analytical grade.

*Determination of a minimum inhibitory concentration (MIC) againstMT*_v_ H_37_R_v_*, MK 235*/*80, MK 6509*/*96 and MA 330*/*80*. Efficiency of the compounds **1a**–**p** and standard drugs (INH, EMB and OFLX, respectively) against given mycobacteria were determined in lyophilized Šula’s semisynthetic medium (Sevac, Prague, Czech Republic) by a dilution-micromethod [20,72,84].

Each tested mycobacterial strain was simultaneously inoculated into Petri plates containing the Šula’s medium for sterility control and growth of the inoculum [84]. All screened molecules were added to this medium as solutions in dimethyl sulfoxide (DMSO; Sigma-Aldrich, Irvine, UK). In the *in vitro* assays, following concentrations of the solutions were used: 1000, 500, 250, 125, 62.5, 32, 16, 8, 4, 2, 1, 0.5 and 0.25 μM, respectively. The inoculated plates kept in microtone bags were incubated at 37 °C. Particular reading was carried out macroscopically on a stand with a bottom magnifying mirror using a magnifying glass.

Growth in the plates [20,72] was evaluated after 7, 14 and 21 days (*MK* 235/80, *MK* 6509/96) or after 14 and 21 days (*MT*_v_ H_37_R_v_, *MA* 330/80).

The value of a minimum inhibitory concentration (*MIC*) was the lowest concentration (on the above concentration scale) of a tested compound, which inhibited growth of the mycobacteria [20,72]. The screening was repeated three times and the *MIC* values (in μM units), reported in Table 6 and Table 7, were the same.

*Determination of a minimum inhibitory concentration (MIC) against MT_a_ H_37_R_a_.* The *mycobacterium* was grown on Middlebrook broth (MB; MiddleBrook Pharmaceuticals, Inc., Westlake, TX, USA), supplemented with Oleic-Albumin-Dextrose-Catalase (OADC) supplement (Difco, Lawrence, KS, USA) and salicylate-derived mycobactin J (2 μg/mL; Allied Monitor Inc., Fayette, MO, USA), an iron-binding siderophore [85,86].

At log phase growth, a culture sample (10 mL) was centrifuged at 15,000 rpm/20 min using a bench top centrifuge MPW-65R (MPW Med Instruments, Warsaw, Poland). Following removal of the supernatant, a pellet was washed in fresh liquid Middlebrook 7H9GC broth (MiddleBrook Pharmaceuticals, Inc.) and re-suspended in a fresh OADC-supplemented MB (10 mL).

Turbidity was adjusted to match the McFarland standard No. 1 [87] containing approximately 3 × 10^8^ Colony Forming Units (CFU) with MB broth. Further 1:20 dilution of the culture was performed in MB broth.

Susceptibility of *MT*_a_ H_37_R_a_ was investigated in a 96-well plate format. In the experiments, sterile deionized water (300 µL) was added to all outer-perimeter wells of the plates to minimize evaporation of the medium in test wells during incubation. Each evaluated compound (100 µL) was incubated with *MT*_a_ H_37_R_a_ (100 µL). Dilutions of each compound were prepared in triplicate and final concentrations varied from 1000 μg/mL to 8 μg/mL. All tested molecules were dissolved in DMSO (Sigma-Aldrich, Irvine, UK), and subsequent dilutions were made in supplemented MB broth. The plates were sealed with parafilm and incubated at 37 °C for 7 days.

Following incubation, 10% addition a water-soluble dye, the alamarBlue reagent (AbD Serotec, Kidlington, UK) was mixed into each well. This resaurin-based reagent served as an oxidation-reduction indicator of metabolic function and cellular health in cell viability bioassays [88].

Absorbance readings at 570 nm and 600 nm were taken, initially for background subtraction and after 24h re-incubation. The subtraction is necessary for strongly colored compounds, where the color may interfere with the interpretation of any color change. For non-interfering compounds, a blue color in a well was interpreted as absence of growth and pink color was scored as growth [73,74].

The minimum inhibitory concentration (*MIC*) was defined as the lowest concentration of a compound, at which no visible bacterial growth was observed. In other words, the *MIC* was the lowest concentration that prevented visual color change from blue to pink. The *MIC* value has been routinely and widely used in bacterial assays and it has been a standard detection limit according to the Clinical and Laboratory Standards Institute [73,74]. Clinically used antimycobacterials INH and CPX were applied as reference drugs. The estimated *MIC* values (in μM units) were provided in Table 6.

*Determination of a minimum inhibitory concentration (MIC) against MKDSM, MS and MM.* A broth dilution micromethod in Middlebrook 7H9 medium (Difco, Lawrence, MO, USA) supplemented with BD BBL Middlebrook OADC Enrichment medium (Becton, Dickinson & Company, Franklin Lakes, NJ, USA) containing 8.5 g NaCl, 50.0 g bovine albumin (fraction V), 20.0 g dextrose, 0.03 g catalase, and 0.6 mL oleic acid [89], respectively, was used to determine the *MIC* values for given strains, as described [75].

Tested phenylcarbamic acid-based molecules **1a**–**p** as well as standard drugs INH and CPX were dissolved in DMSO (Sigma-Aldrich, Irvine, UK) and final concentration of DMSO did not exceed 2.5% of the total solution composition. The final concentrations, varying from 256 μg/mL to 0.125 μg/mL, were obtained by a two-fold serial dilution of a stock solution in a microtiter plate with sterile medium.

Bacterial inocula were prepared by transferring colonies from culture into sterile water. Cell density was adjusted to the 0.5 McFarland units [87] using the cell density meter Densi-La-Meter (LIAP, Riga, Latvia). Final inoculum was made by 1:1000 dilution of the suspension with sterile water. Drug-free controls, sterility controls and controls consisted of medium and DMSO alone were included. Results were determined visually after static incubation in darkness in aerobic atmosphere for: (i) 3 days at 37 °C in a case of *MS*; (ii) 7 days at 37 °C (*MK* DSM) and (iii) 21 days at 28 °C (*MM*), respectively.

The *MIC* parameter was defined as the lowest concentration of a compound, at which no visible bacterial growth was observed. The *MIC* value has been routinely and widely used in bacterial assays considering it a standard detection limit according to the Clinical and Laboratory Standards Institute [73,74]. Presently observed *MIC* values (in μM units) are provided in Table 6 and Table 7.

### 3.5. Calculations and Statistical Analyses

Regression equations of relevant fitting procedures and their statistical characteristics were calculated and visualized by the Origin Pro *ver.* 9.0.0 SR2 software (OriginLab Corporation, Northampton, MA, USA).

In a current research, those statistical parameters were calculated: number of points (number of cases; *n*), degrees of freedom (*DF*), reduced chi-square (*χ*,^2^_red_), residual sum of squares (*RSS*), correlation coefficient (*R*), adjusted coefficient of determination (*Adj. R*^2^), root mean squared error (standard deviation; *RMSE*), norm of residuals (*NR*), Fisher’s significance ratio (Fisher’s *F*-test; *F*) and probability of obtaining *F* Ratio (significance of a whole model; *Prob > F*), respectively.

The analyses were also focused on indication of a significance level of calculated *F* Ratio as follows: * (one star)—statistically significant; ** (two stars)—statistically very significant; *** (three stars)—statistically extremely significant. Detailed information regarding given statistical descriptors could be found in a research paper [47].

The chemometric principal component analysis (PCA) tool was used to explore relationships between (i) experimental and *in silico* lipophilicity descriptors, (ii) *in vitro* efficiency against all tested mycobacterial strains as well as (iii) physicochemical descriptors and *in vitro* biological activities of the compounds **1a**–**p**. The PCA is a mathematical algorithm that reduces the dimensionality of the data while retaining most of variation in the data set. It accomplishes this reduction by identifying directions, called principal components (PCs), along which the variation in the data is maximal. By using a few components, each sample can be represented by relatively few numbers instead of by values for thousands of variables. Samples can then be plotted, making it possible to visually assess similarities and differences between samples and determine whether samples can be grouped [90].

The PCA was performed by the XLSTAT *ver.* 2016.02.28451 software (Addinsoft, New York, NY, USA), a cloud-based statistical application for statistics and data analyses, which worked as an add-on to the Microsoft Excel *ver.* 2013 software (Microsoft Corp., Redmont, WA, USA).

## 4. Conclusions

In summary, determination of physicochemical properties and *in vitro* antimymycobacterial screening in connection with *SAR* analyses of 1-[2-[({[2-/3-(alkoxy)phenyl]amino}carbonyl)oxy]-3-(dipropylammonio)propyl]pyrrolidinium oxalates (**1a**–**d**)/dichlorides (**1e**–**h**) and 1-[2-[({[2-/3-(alk-oxy)phenyl]amino}carbonyl)oxy]-3-(dipropylammonio)propyl]azepanium oxalates (**1i**–**l**)/di-chlorides (**1m**–**p**; alkoxy = butoxy to heptyloxy) were considered main objectives of the research.

All compounds **1a**–**p** in aqueous solutions (*c* = 2.0 × 10^−3^ M) were able to decrease surface tension of water (γ = 0.07259 N/m) at 21 °C and their γ varied from 0.05692 N/m (**1p**) to 0.06464 N/m (**1a**). Relationships between number of carbon atoms forming their side chain *R* (*n*_c_) and γ values (in N/m units) were described most precisely by statistically significant or very significant models based on polynomial functions of 2^nd^ order within all homological subsets **1a**–**d**, **1e**–**h**, **1i**–**l** and **1m**–**p**.

Electronic properties of the molecules **1a**–**p** were characterized by logarithms of molar absorption coefficients (log *ε*) of their methanolic solutions (*c* = 8.0 × 10^−5^ M) investigated in the UV/Vis region of an electromagnetic spectrum. The solutions showed three absorption maxima in a near ultraviolet (quarz) region of the spectrum between 200 nm and 400 nm. The maxima were found at *λ*_1_ = 208–210 nm (second local excitation band), *λ*_2 (Ch-T)_ = 236–238 nm (charge-transfer band) and *λ*_3_ = 278–280 nm (first local excitation band), respectively. There was observed nor linear neither quasi-parabolic relationship between *n*_c_ and log *ε*_2 (Ch-T)_, as the maximum being the most sensitive to differences in electronic environment of a phenylcarbamoyloxy moiety due to different position and length of a substituent *R*.

The log *k*_w_ values, as descriptors characterizing lipophilic properties of **1a**–**p**, were extrapolated from intercepts of statistically extremely significant linear relationships between log *k* parameters and volume fractions of MeOH modifier (*ϕ*_M_) using the Snyder–Soczewiński solvent strength model. These log *k*_w_ values were in accordance with an elution order and hydrophobicity of the molecules **1a**–**p** and varied from 3.7688 (**1a**) to 6.1749 (**1p**). Increase in length of their side chain *R* led to higher *S*, as a function of molecular structure parameters, within all homological sets. The compounds containing an azepanium moiety showed higher *S* than those with a pyrrolidium group presuming same position and length of *R*. The calculated *S* parameters varied from 3.7788 (**1a**) to 6.2672 (**1p**). A statistically extremely significant relationship between the log *k*_w_ and *S* values was found.

The experimental log *k*_w_ dataset was studied together with computational logarithms of partition coefficients (log *P*). The log *P* parameters of non-protonated bases **9′a**–**p** were calculated for the octan-1-ol/water partitioning system using the Ghose and Crippen′s log *P*_Cr_, Viswanadhan′s log *P*_V_, Broto’s log *P*_B_, Leo’s CLOGP 4.0, Wang’s XLOGP 2.0, Cheng’s XLOGP 3.0, Moriguchi’s MLOGP, Sander’s (Actelion’s) ACLOGP, Molinspiration’s miLogP 2.2, ALOGP, SILICOS-IT and Tetko´s ALOGPs 2.1 method as well. Similarities and differences between log *k*_w_ and *in silico* descriptors were analyzed by unscaled principal component analysis (PCA). There was confirmed high sensitivity of PCA for detecting of small differerences between analyzed positional isomers, which given *in silico* methods could not perform with satisfactory visibility. The log *P*_I-IT_, XLOGP 2.0 and XLOGP 3.0 descriptors might be used to characterize lipophilic properties of investigated salts and bases, especially if they contained highly lipophilic side chain *R*.

The compounds **1a**–**p** were *in vitro* screened against *Mycobacterium tuberculosis* CNCTC My 331/88 (identical with H_37_R_v_; abbreviation used: *MT*_v_ H_37_R_v_), *M. tuberculosis* H_37_R_a_ ATCC 25177 (*MT*_a_ H_37_R_a_), *M. kansasii* CNCTC My 235/80 (*MK* 235/80), the *M. kansasii* 6509/96 clinical isolate (*MK* 6509/96), *M. kansasii* DSM 44162 (*MK* DSM), *M. avium* CNCTC My 330/80 (*MA* 330/80), *M. smegmatis* ATCC 700084 (*MS*) and *M. marinum* CAMP 5644 (*MM*), respectively. *In vitro* susceptibility of given mycobacteria to clinically used reference drugs isoniazid (INH), ethambutol (EMB), ofloxacin (OFLX) or ciprofloxacin (CPX) was tested as well.

A very unique aspect of a present research was that many compounds from the set **1a**–**p** were *in vitro* efficient against almost all tested mycobacterial strains. The compounds **1h** and **1p** were the most active practically against the whole spectrum of tested mycobacteria (*MIC* ≤ 8 μM), excluding *MA* 330/80.

All 3-alkoxy substituted molecules **1e**–**h** and **1m**–**p** were more efficient against *MT*_a_ H_37_R_a_ (*MIC* = 3.7–8.1 μM) than INH or CPX. The dibasic compounds also inhibited growth of *MK* DSM more effectively (*MIC* = 1.9–16.2 μM) compared to INH. Moreover, the derivatives **1g**, **1h** and **1n**–**p** showed approximately equal activity against *MA* 330/80 (*MIC* = 16 μM) as EMB. Regarding *MT*_v_ H_37_R_v_, however, none of the molecules **1a**–**p** showed similar ability to fight given *mycobacterium* as INH. Comparable activity against *MK* 6509/96 was found for the substances **1h**, **1n**–**p** (*MIC* = 4 μM and 8 μM) and INH. If concerning the 2-alkoxy substituted compounds **1a**–**d** and **1i**–**l**, the derivative **1d** was more promising against *MT*_a_ H_37_R_a_ (*MIC* = 12.5 μM) and *MK* DSM (*MIC* = 6.2 μM) than INH. The INH standard was also less effective against *MS* or *MM* than practically all tested phenylcarbamic acid-based substances, from which were **1j** (*MIC* = 6.2 μM; anti-*MS*) and **1h** (*MIC* < 3.7 μM; anti-*MM*) the most efficient.

The chemometric PCA approach as well as fitting procedures, supported by relevant equations and adequate statistical analyses, were used to comprehensively investigate *SAR* of the derivatives **1a**–**p**. It seemed that ability to decrease surface tension of water was slightly more important than increase in lipophilicity of a pyrrolidinium moiety-containing compounds **1a**–**h** in order to improve their efficiency against both *MT*_v_ H_37_R_v_ and *MA* 330/80. The compounds with γ varying from 0.05692 N/m (**1p**) to 0.06154 N/m (**1n**) showed comparable potential to act against *MA* 330/80 (21-d). Further increase in γ, i.e., minor ability to decrease surface tension of water, resulted in sharp decrease in their antimycobacterial efficiency. If attention was turned to the 3-alkoxy substituted molecules **1e**–**h** and **1m**–**p**, increase in their log *k*_w_ up to approximately 5.1000 led to sharp increase in activity against *MA* 330/80 (21-d). Further increase in lipophilicity beyond this border did not result in higher antimycobacterial activity of **1n** (log *k*_w_ = 5.2087), **1h** (log *k*_w_ = 5.5384), **1o** (log *k*_w_ = 5.6569) or **1p** (log *k*_w_ = 6.1749).

Increase in lipophilicity of the molecules **1a**–**h** was more important factor, which positively influenced their efficiency against *MK* 235/80, *MK* 6509/96 and *MK* DSM, compared to surface properties. Conversely, incease in lipophilicity over ‘a certain value’ (log *k*_w_ of approximately 5.2000) together with different size and steric properties of a salt-forming group (**1i**–**p**) would not be a guaranty of improvement in antimycobacterial activity.

There was observed a statistically very significant model between log *ε*_2 (Ch-T)_ and log (1/*MIC* [M]) connected with 3-d *in vitro* evaluation of the compounds **1c**–**h** against *MS*. It was found that activity increased with increasing log *ε*_2 (Ch-T)_, reached a maximum if the independent variable was between 4.24 and 4.27 (**1e**, **1f** and **1h**) and then decreased with further increase in the log *ε*_2 (Ch-T)_ values.

Overall, the results of current *in vitro* biological screening and *SAR* investigations of the molecules **1a**–**p** might reveal new horizons in discovery of very effective ‘non-traditional’ antimycobacterial agents based on a phenylcarbamic acid structural scaffold.

## Figures and Tables

**Table 1:** Chemical structure of evaluated compounds **1a**–**p**, their surface tension γ (relative surface activity; in N/m units), wavelengths of observed absorption maxima (*λ*_1_, *λ*_2 (Ch-T)_, *λ*_3_) and logarithms of molar absorption coefficients (log *ε*_1_, log *ε*_2 (Ch-T)_, log *ε*_3_) of compounds’ methanolic solutions (*c* = 8.0 × 10^−5^ M), which were investigated in the UV/Vis region of an electromagnetic spectrum.

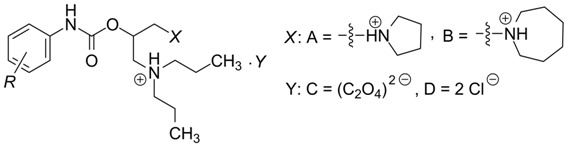										
Comp.	*R*	*X*	*Y*	γ (N/m)	*λ*_1_	log *ε*_1_	*λ*_2 (Ch-T)_	^1^ log *ε*_2 (Ch-T)_	*λ*_3_	log *ε*_3_
**1a**	2-OC_4_H_9_	A	C	0.06464	208	4.51	236	4.19	280	3.63
**1b**	2-OC_5_H_11_	A	C	0.06366	208	4.39	236	4.05	280	3.51
**1c**	2-OC_6_H_13_	A	C	0.06222	208	4.58	238	4.52	278	3.49
**1d**	2-OC_7_H_15_	A	C	0.05985	208	4.43	236	4.08	280	3.55
**1e**	3-OC_4_H_9_	A	D	0.06316	210	4.65	238	4.24	279	3.59
**1f**	3-OC_5_H_11_	A	D	0.06285	210	4.55	237	4.27	279	3.62
**1g**	3-OC_6_H_13_	A	D	0.06105	210	4.54	237	4.13	279	3.48
**1h**	3-OC_7_H_15_	A	D	0.05786	210	4.66	237	4.27	279	3.63
**1i**	2-OC_4_H_9_	B	C	0.06302	208	4.44	236	4.08	280	3.57
**1j**	2-OC_5_H_11_	B	C	0.06206	208	4.54	236	4.22	280	3.71
**1k**	2-OC_6_H_13_	B	C	0.06065	208	4.42	236	4.10	280	3.57
**1l**	2-OC_7_H_15_	B	C	0.05853	208	4.44	236	4.14	280	3.59
**1m**	3-OC_4_H_9_	B	D	0.06298	210	4.52	238	4.09	279	3.44
**1n**	3-OC_5_H_11_	B	D	0.06154	210	4.56	238	4.18	279	3.52
**1o**	3-OC_6_H_13_	B	D	0.05925	210	4.52	238	4.01	279	3.37
**1p**	3-OC_7_H_15_	B	D	0.05692	210	4.65	238	4.20	279	3.56

^1^ log *ε*_2 (Ch-T)_, Logarithms of molar absorption coefficients observed at the charge-transfer absorption maximum *λ*_2 (Ch-T)_ = 236–238 nm.

**Table 2:** Retention times *t*_r_ (RP-HPLC) and lipophilicity indices log *k* (RP-HPLC) of the compounds **1a**–**p** estimated in the mobile phases with a various volume ratio (*v*/*v*) of a methanol (MeOH) organic modifier and water.

Comp.	Mobile Phase MeOH/Water (*v*/*v*)									
	80:20		85:15		90:10		95:5		Pure MeOH	
	*t*_r_ (min)	log *k*	*t*_r_ (min)	log *k*	*t*_r_ (min)	log *k*	*t*_r_ (min)	log *k*	*t*_r_ (min)	log *k*
**1a**	13.883	0.7165	10.694	0.5756	7.830	0.3958	5.766	0.2008	4.224	−0.0403
**1b**	17.200	0.8254	12.647	0.6656	9.449	0.5064	6.514	0.2841	4.526	0.0203
**1c**	25.694	1.0206	16.950	0.8162	11.292	0.6053	7.431	0.3683	5.246	0.1287
**1d**	31.000	1.1092	19.600	0.8882	13.553	0.7022	7.892	0.4052	5.385	0.1573
**1e**	10.330	0.5584	6.917	0.3183	5.400	0.1478	3.822	−0.1454	3.329	−0.2956
**1f**	12.400	0.6574	9.200	0.4911	6.280	0.2546	4.022	−0.0941	3.340	−0.2913
**1g**	17.200	0.8254	11.402	0.6104	7.483	0.3679	5.153	0.1182	3.583	−0.2067
**1h**	22.003	0.9521	13.701	0.7159	8.532	0.4565	5.788	0.2092	3.556	−0.2153
**1i**	24.020	0.9881	16.917	0.8153	11.691	0.6240	7.765	0.3954	5.127	0.1205
**1j**	29.525	1.0863	20.021	0.8981	13.060	0.6828	8.520	0.4509	5.295	0.1449
**1k**	47.108	1.3023	27.001	1.0424	16.533	0.8038	9.641	0.5221	6.938	0.3303
**1l**	70.012	1.4831	38.800	1.2152	22.636	0.9619	11.670	0.6271	7.756	0.3996
**1m**	15.731	0.7805	9.218	0.4922	6.420	0.2695	4.685	0.0424	3.519	−0.2275
**1n**	22.183	0.9502	12.370	0.6542	8.156	0.4205	4.995	0.0941	3.947	−0.1047
**1o**	29.174	1.0807	13.651	0.7059	9.634	0.5174	5.095	0.1095	4.127	−0.0618
**1p**	33.719	1.1484	17.129	0.8215	11.332	0.6072	5.801	0.2051	3.925	−0.1102

**Table 3:** Extrapolated log *k*_w_ parameters (RP-HPLC) of analyzed molecules **1a**–**p** and values of statistical descriptors (*χ*^2^_red_, *RSS*, *R*, *Adj. R*^2^, *RMSE*, *NR*, *F* and *Prob > F*), which characterized a linear relationship between the log *k* and *ϕ*_M_ values for a particular compound. The *ϕ*_M_ parameter was a volume fraction of MeOH in the isocratic elution RP-HPLC.

Comp.	log *k*_w_	^1^ *S*	^2^ *χ* ^2^_red_	^3^ *RSS*	^4^ *R*	^5^ *Adj. R* ^2^	^6^ *RMSE*	^7^ *NR*	^8^ *F*	^9^ *Prob > F*
**1a**	3.7688	3.7768	0.0011	0.0034	0.9953	0.9875	0.0335	0.0580	317.64	0.0001 ***
**1b**	4.0454	3.9834	0.0018	0.0055	0.9931	0.9817	0.0429	0.0744	215.17	0.0007 ***
**1c**	4.6049	4.4634	0.0002	0.0007	0.9993	0.9982	0.0152	0.0263	2155.37	0.0001 ***
**1d**	4.9487	4.7736	0.0011	0.0033	0.9971	0.9923	0.0332	0.0574	518.15	0.0002 ***
**1e**	4.0258	4.3434	0.0013	0.0038	0.9960	0.9894	0.0354	0.0614	375.47	0.0003 ***
**1f**	4.6722	4.9652	0.0028	0.0084	0.9932	0.9821	0.0530	0.0917	219.79	0.0007 ***
**1g**	4.9446	5.1128	0.0013	0.0040	0.9969	0.9919	0.0366	0.0634	487.75	0.0002 ***
**1h**	5.5384	5.6830	0.0042	0.0126	0.9923	0.9795	0.0648	0.1122	192.51	0.0008 ***
**1i**	4.4679	4.3102	0.0014	0.0042	0.9955	0.9879	0.0376	0.0652	328.27	0.0004 ***
**1j**	4.8466	4.6600	0.0016	0.0048	0.9956	0.9882	0.0401	0.0695	337.19	0.0004 ***
**1k**	5.2359	4.9286	0.0005	0.0017	0.9987	0.9964	0.0234	0.0406	1106.57	0.0001 ***
**1l**	5.8966	5.5102	0.0007	0.0020	0.9988	0.9965	0.0259	0.0449	1127.92	0.0001 ***
**1m**	4.7099	4.9316	0.0004	0.0013	0.9990	0.9972	0.0205	0.0356	1442.73	0.0001 ***
**1n**	5.2087	5.3398	0.0011	0.0032	0.9978	0.9941	0.0325	0.0564	673.05	0.0001 ***
**1o**	5.6569	5.7628	0.0045	0.0134	0.9920	0.9789	0.0668	0.1157	186.23	0.0009 ***
**1p**	6.1749	6.2672	0.0022	0.0067	0.9966	0.9909	0.0474	0.0820	438.042	0.0001 ***

^1^*S*, Slope; ^2^*χ*^2^_red_, reduced chi-square; ^3^*RSS*, residual sum of squares; ^4^*R*, correlation coefficient; ^5^*Adj. R*^2^, adjusted coefficient of determination; ^6^*RMSE*, root mean squared error (standard deviation); ^7^*NR*, norm of residuals; ^8^*F*, Fisher’s significance ratio (Fisher’s *F*-test); ^9^*Prob > F*, probability of obtaining the *F* Ratio (significance of a whole model). Indication of a significance level of the *F* Ratio was as follows: *** (three stars), extremely significant.

**Table 4:** Values of logarithms of partition coefficients of basic (non-protonated) compounds **9′a**–**p** predicted for the octan-1-ol/water partitioning system by the Ghose and Crippen′s approach (log *P*_Cr_), Viswanadhan′s principle (log *P*_V_), Broto′s algorithm (log *P*_B_) and CLOGP method (CLOGP 4.0), respectively, as well as by both XLOGP approaches (XLOGP 2.0 and XLOGP 3.0).

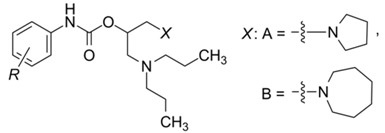								
Comp.	*R*	*X*	log *P*_Cr_	log *P*_V_	log *P*_B_	CLOGP 4.0	XLOGP 2.0	XLOGP 3.0
**9′a**	2-OC_4_H_9_	A	4.59	4.45	4.51	6.23	4.52	5.14
**9′b**	2-OC_5_H_11_	A	5.00	4.85	4.97	6.76	5.09	5.68
**9′c**	2-OC_6_H_13_	A	5.42	5.24	5.42	7.29	5.66	6.22
**9′d**	2-OC_7_H_15_	A	5.84	5.64	5.88	7.82	6.23	6.77
**9′e**	3-OC_4_H_9_	A	4.59	4.45	4.51	6.23	4.52	5.14
**9′f**	3-OC_5_H_11_	A	5.00	4.85	4.97	6.76	5.09	5.68
**9′g**	3-OC_6_H_13_	A	5.42	5.24	5.42	7.29	5.66	6.22
**9′h**	3-OC_7_H_15_	A	5.84	5.64	5.88	7.82	6.23	6.77
**9′i**	2-OC_4_H_9_	B	5.42	5.24	5.42	7.35	5.24	5.86
**9′j**	2-OC_5_H_11_	B	5.84	5.64	5.88	7.88	5.81	6.40
**9′k**	2-OC_6_H_13_	B	6.26	6.04	6.33	8.41	6.38	6.84
**9′l**	2-OC_7_H_15_	B	6.67	6.43	6.79	8.94	6.95	7.48
**9′m**	3-OC_4_H_9_	B	5.42	5.24	5.42	7.35	5.24	5.86
**9′n**	3-OC_5_H_11_	B	5.84	5.64	5.88	7.88	5.81	6.40
**9′o**	3-OC_6_H_13_	B	6.26	6.04	6.33	8.41	6.38	6.84
**9′p**	3-OC_7_H_15_	B	6.67	6.43	6.79	8.94	6.95	7.48

**Table 5:** Values of logarithms of partition coefficients of basic (non-protonated) compounds **9′a**–**p** predicted for the octan-1-ol/water partitioning system by the MLOGP, ACLOGP, miLogP 2.2, ALOGP, SILICOS-IT (log *P*_S-IT_) and ALOGPs 2.1 method, respectively.

Comp.	MLOGP	ACLOGP	miLogP 2.2	ALOGP	log *P*_S-IT_	ALOGPs 2.1
**9′a**	2.75	5.05	5.30	5.42	4.27	5.18
**9′b**	2.96	5.52	5.80	5.87	4.68	5.57
**9′c**	3.16	5.98	6.32	6.33	5.10	5.93
**9′d**	3.36	6.45	6.81	6.79	5.53	6.25
**9′e**	2.75	5.05	5.32	5.42	4.27	5.21
**9′f**	2.96	5.52	5.83	5.87	4.68	5.61
**9′g**	3.16	5.98	6.33	6.33	5.10	5.95
**9′h**	3.36	6.45	6.84	6.79	5.53	6.32
**9′i**	3.16	5.69	6.31	6.33	4.78	5.93
**9′j**	3.36	6.16	6.81	6.79	5.20	6.23
**9′k**	3.56	6.62	7.32	7.24	5.62	6.54
**9′l**	3.76	7.08	7.82	7.70	6.04	6.77
**9′m**	3.16	5.69	6.33	6.33	4.78	5.97
**9′n**	3.36	6.16	6.84	6.79	5.20	6.27
**9′o**	3.56	6.62	7.34	7.24	5.62	6.59
**9′p**	3.76	7.08	7.85	7.70	6.04	6.83

**Table 6:** The *in vitro* activity (*MIC* values in μM units) of investigated compounds **1a**–**p** and reference drugs isoniazid (INH), ethambutol (EMB), ofloxacin (OFLX) and ciprofloxacin (CPX) against *M. tuberculosis* CNCTC My 331/88 (*M. tuberculosis* H_37_R_v_; *MT*_v_ H_37_R_v_), *M. tuberculosis* H_37_R_a_ ATCC 25177 (*MT*_a_ H_37_R_a_), *M. kansasii* CNCTC My 235/80 (*MK* 235/80), *M. kansasii* 6509/96 (*MK* 6509/96) and *M. kansasii* DSM 44162 (*MK* DSM), respectively.

Comp.	*MIC* [μM]									
	*MT*_v_ H_37_R_v_		*MT*_a_ H_37_R_a_	*MK* 235/80			*MK* 6509/96			*MK* DSM
	^1^ 14-d	^2^ 21-d	^3^ 7-d	7-d	14-d	21-d	7-d	14-d	21-d	7-d
**1a**	32	32	213	62.5	62.5	62.5	32	62.5	62.5	53
**1b**	16	32	52	32	32	62.5	32	32	62.5	26.1
**1c**	16	16	25	32	32	32	16	16	16	12.7
**1d**	8	8	12.5	16	16	32	8	16	16	6.2
**1e**	16	32	8.1	32	32	32	16	16	32	16.2
**1f**	16	16	<3.9	16	16	32	8	16	16	7.9
**1g**	8	16	<3.8	16	16	16	8	8	16	3.8
**1h**	4	8	<3.7	8	8	8	4	4	8	1.9
**1i**	16	32	203	32	62.5	62.5	16	32	32	51
**1j**	16	32	199	16	32	62.5	16	32	32	50
**1k**	8	16	195	16	32	62.5	16	32	32	98
**1l**	4	8	96	16	32	32	16	16	32	382
**1m**	8	16	15.4	16	16	32	8	16	16	7.7
**1n**	4	8	7.5	8	8	8	4	8	8	7.5
**1o**	8	16	7.3	8	16	16	4	8	8	3.6
**1p**	4	8	7.1	8	8	8	4	8	8	3.6
**INH**	0.5	1	36.5	>250	>250	>250	2	4	8	29.2
**EMB**	1	2	–	–	–	–	1	2	2	–
**OFLX**	1	2	–	0.5	1	1	0.5	0.5	1	–
**CPX**	–	–	48.3	–	–	–	–	–	–	3.0

^1^ 14-d, 14-Day cultivation; ^2^ 21-d, 21-day cultivation; ^3^ 7-d, 7-day cultivation. The most promising values of compounds *in vitro* antimycobacterial activity (*MIC* ≤ 8 μM) were indicated in gray cells of Table 6.

**Table 7:** The *in vitro* activity (*MIC* values in μM units) of investigated compounds **1a**–**p** and reference drugs isoniazid (INH), ethambutol (EMB), ofloxacin (OFLX) and ciprofloxacin (CPX) against *M. avium* CNCTC My 330/80 (*MA* 330/80), *M. smegmatis* ATCC 700084 (*MS*) and *M. marinum* CAMP 5644 (*MM*), respectively.

Comp.	*MIC* [μM]			
	*MA* 330/80		*MS*	*MM*
	^1^ 14-d	^2^ 21-d	^3^ 3-d	21-d
**1a**	125	125	213	106
**1b**	62.5	125	104	52
**1c**	32	62.5	51	25.5
**1d**	32	32	24.9	12.5
**1e**	62.5	62.5	16.2	32.5
**1f**	32	32	15.8	<3.9
**1g**	16	16	15.4	<3.8
**1h**	8	16	15.0	<3.7
**1i**	62.5	125	203	51
**1j**	62.5	125	6.2	50
**1k**	32	62.5	390	98
**1l**	32	62.5	382	191
**1m**	16	32	30.7	15.4
**1n**	16	16	15.0	15.0
**1o**	16	16	7.3	7.3
**1p**	16	16	7.1	7.1
**INH**	> 250	> 250	117	467
**EMB**	16	16	–	–
**OFLX**	32	62.5	–	–
**CPX**	–	–	0.4	0.8

^1^ 14-d, 14-Day cultivation; ^2^ 21-d, 21-day cultivation; ^3^ 3-d, 3-day cultivation. The most promising values of compounds´ *in vitro* antimycobacterial activity (*MIC* ≤ 8 μM) were indicated in gray cells of Table 7.

## Supplementary Materials

The supplementary materials are available online.

## Abbreviations

The following abbreviations are used in this manuscript:

2-PrOH	Propan-2-ol
3-d	3-Day cultivation
7-d	7-Day cultivation
14-d	14-Day cultivation
21-d	21-Day cultivation
*λ*_e_	Eigenvalues (principal component analysis)
*χ*,^2^_red_	Reduced chi-square (statistical analysis)
*ϕ*_M_	Volume fraction of a mobile phase modifier (RP-HPLC)
CPX	Ciprofloxacin
*Adj. R* ^2^	Adjusted coefficient of determination (statistical analysis)
DEE	Diethyl ether
*DF*	Degrees of freedom (statistical analysis)
DMSO	Dimethyl sulfoxide
EtOH	Ethanol
EMB	Ethambutol
*F*	Fisher’s *F*-test (Fisher’s significance ratio; statistical analysis)
INH	Isoniazid
IR	Infrared
*k*	Retention (capacity) factor (RP-HPLC)
LA/LAs	Local anesthetic/local anesthetics
log *k*_w_	Lipophilicity index; values extrapolated from intercepts of a linear relationship between the logarithm of retention factor *k* (log *k*) and volume fraction of a mobile phase modifier (*ϕ*_M_; RP-HPLC)
MeOH	Methanol
*MA*	*Mycobacterium avium*
*MIC*	Minimum inhibitory concentration (in μΜ units)
*MK*	*Mycobacterium kansasii*
*MM*	*Mycobacterium marinum*
*MS*	*Mycobacterium smegmatis*
*MT*	*Mycobacterium tuberculosis*
*n*_c_	Number of carbon atoms forming an 2-/3-alkoxy side chain (*R*)
*NR*	Norm of residuals (statistical analysis)
OFLX	Ofloxacin
PC	Principal Component (principal component analysis)
PCA/PCAs	Principal component analysis/principal component analyses
*Prob > F*	Probability of obtaining the *F* Ratio (statistical analysis)
*RMSE*	Root mean squared error (standard deviation; statistical analysis)
*RSS*	Residual sum of squares (statistical analysis)
*S*	Slope (RP-HPLC)
*SAR*/*SARs*	Structure–activity relationship/Structure–activity relationships
*t*_r_	Retention time of a compound (RP-HPLC)
