# Supplementary material for: Dibasic Derivatives of Phenylcarbamic Acid against Mycobacterial Strains: Old Drugs and New Tricks?
Source: Molecules. 2018 Sep 28;23(10):2493. doi: 10.3390/molecules23102493 (PMC6222509; doi:10.3390/molecules23102493)
Supplement: Supplementary file 1 [file molecules-23-02493-s001.pdf]

Supplementary Materials

Dibasic Derivatives of Phenylcarbamic Acid Against Mycobacterial Strains: Old Drugs and New Tricks?

Ivan Malík [1,*], Jozef Csöllei [2], Ivan Solovič [3,4], Šárka Pospíšilová [1], Hana Michnová [1], Josef Jampílek [1], Alois Čížek [5], Iva Kapustíková [1], Jana Čurillová [1], Mária Pecháčová [1], Jiřina Stolaříková [6], Daniel Pecher [7,8] and Michal Oravec [9]

[1] Department of Pharmaceutical Chemistry, Faculty of Pharmacy, Comenius University in Bratislava, Odbojárov 10, Bratislava SK-832 32, Slovak Republic; malikivan001@gmail.com (I.M.); sharka.pospisilova@gmail.com (Š.P.); michnova.hana@gmail.com (H.M.); josef.jampilek@gmail.com (J.J.); kapustikova@fpharm.uniba.sk (I.K.); curilova2@uniba.sk (J.Č.); mariapech901@gmail.com (M.P.)

[2] Department of Chemical Drugs, Faculty of Pharmacy, University of Veterinary and Pharmaceutical Sciences in Brno, Palackého 1946/1, Brno CZ-612 42, Czech Republic; csolleij@vfu.cz (J.Cs.)

[3] Clinic for Tuberculosis and Lung Diseases, National Institute for Tuberculosis, Lung Diseases and Thoracic Surgery, Vyšné Hágy, Vysoké Tatry SK-059 84, Slovak Republic; solovic@hagy.sk (I.S.)

[4] Department of Public Health, Faculty of Health, Catholic University in Ružomberok, Hrabovská cesta 1A, Ružomberok SK-034 01, Slovak Republic; ivan.solovic@ku.sk (I.S.)

[5] Clinic for Department of Infectious Diseases and Microbiology, Faculty of Veterinary Medicine, University of Veterinary and Pharmaceutical Sciences, Palackého 1946/1, Brno CZ-612 42, Czech Republic; cizeka@vfu.cz (A.Č.)

[6] Laboratory for Mycobacterial Diagnostics and Tuberculosis, Regional Institute of Public Health, Partyzánské náměstí 7, Ostrava CZ-702 00, Czech Republic; Jirina.Stolarikova@zu.cz (J.S.)

[7] Department of Pharmaceutical Analysis and Nuclear Pharmacy, Faculty of Pharmacy, Comenius University in Bratislava, Odbojárov 10, Bratislava SK-832 32, Slovak Republic; pecher1@uniba.sk (D.P.)

[8] Toxicological and Antidoping Center, Faculty of Pharmacy, Comenius University in Bratislava, Odbojárov 10, Bratislava SK-832 32, Slovak Republic; pecher1@uniba.sk (D.P.)

[9] Global Change Research Institute CAS, Belidla 986/4a, Brno CZ-603 00, Czech Republic; oravec.m@czechglobe.cz (M.O.)

* Correspondence: malikivan001@gmail.com; Tel.: +421-2-501-117-227

Synthesis of Analyzed Compounds

Presently investigated 1-[2-[({[2-/3-(alkoxy)phenyl]amino}carbonyl)oxy]-3-(dipropylammonio)-propyl]pyrrolidinium oxalates (**1a–d**)/dichlorides (**1e–h**) as well as 1-[2-[({[2-/3-(alkoxy)phenyl]-amino}carbonyl)oxy]-3-(dipropylammonio)propyl]azepanium oxalates (**1i–l**)/dichlorides (**1m–p**; alkoxy = butoxy to heptyloxy) were prepared by multi step pathways (Scheme) using 2-aminophenol (**1´a**) and 3-aminophenol (**1´b**), respectively, as starting compounds [21,27–29].

Procedures for syntheses of particular reaction intermediates **2´a**, **2´b**, **3´a–h**, **4´a–h**, **5´a–h**, **7´**, **8´a**, **8´b** and **9´a–p** as well as final compounds **1a–p** were provided in next sections of the *supplementum*.

General Procedure For the Preparation of N-(2-/3-Hydroxyphenyl)ethanamides

Into a suspension of 2-aminophenol (**1´a**; CAS Registry Number 95-55-6; 502.00 g, 4.60 mol) or 3-aminophenol (**1´b**; CAS Registry Number 591-27-5; 502.00 g, 4.60 mol) in an aqueous solution of acetic acid, acetanhydride (CAS Registry Number 108-24-7) was added dropwisely (408.36 g, 4.00 mol; Scheme) over 30 min. These mixtures were stirred (30 min) at room temperature (r.t.) and heated (1h) in a water bath. After heating, the reaction systems were kept 12h to cool to r.t., both crude *N*-(2-hydroxyphenyl)ethanamide and *N*-(3-hydroxyphenyl)ethanamide were isolated, washed with water under reduced pressure, air-dried and precipitates were crystallized from a mixture of water and methanol (5:1 (*v*/*v*)) to afford very slightly brownish crystalline solids. Yields (in percentages), melting point values as well as infrared (IR) spectra of both *N*-(2-hydroxy-phenyl)ethanamide (**2´a**) and *N*-(3-hydroxyphenyl)ethanamide (**2´b**) were published in a paper [27].

General Procedure For the Preparation of N-(2-/3-Alkoxyphenyl)ethanamides

In a first step, basic metal alkanoate was prepared, i.e., metallic sodium (22.99 g, 1.00 mol) was very carefully dissolved in anhydrous ethanol (EtOH; 500 mL) under reflux. A solution of *N*-(2-hy-droxyphenyl)ethanamide (**2´a**; 151.17 g, 1.00 mol) or *N*-(3-hydroxyphenyl)ethanamide (**2´b**; 151.17 g, 1.00 mol) in anhydrous EtOH (500 mL) was carefully added to the sodium ethanoate solution.

After mixing, 1-bromoalkane (1.00 mol), i.e., 1-bromobutane (CAS Registry Number 109-65-9; 137.02 g), 1-bromopentane (CAS Registry Number 110-53-2; 151.05 g), 1-bromohexane (CAS Registry Number 111-25-1; 165.07 g) and 1-bromoheptane (CAS Registry Number 629-04-9; 179.10 g), respectively, was added. Particular reactions were allowed to stand (12h) to cool to r.t. and heated up to reflux (3h). A precipitated sodium bromide was filtered off, solvent was removed *in vacuo* and cold distilled water (2000 mL) was added to the residuum.

Crude *N*-(2-/3-alkoxyphenyl)ethanamides (**3´a–h**; alkoxy = butoxy to heptyloxy) were isolated, washed with an aqueous sodium hydroxide solution and finally with water to a neutral reaction [27]. The intermediates **3´a–h** (Scheme) were crystallized from a mixture of water and EtOH (3:1, (*v*/*v*)). Yields (in percentages), melting point values as well as IR spectra of the compounds **3´a–h** were published in a paper [27].

General Procedure For the Preparation of of 2-/3-Alkoxyanilines

N-(2-/3-Alkoxyphenyl)ethanamides (**3´a–h**; alkoxy = butoxy to heptyloxy; 1.00 mol), i.e., **3´a**, **3´e** (both 207.27 g), **3´b**, **3´f** (221.30 g), **3´c**, **3´g** (235.32 g), **3´d** and **3´h** (249.35 g), respectively, were suspended in 18% hydrochloric acid (400 mL) and heated up to reflux (2h) in an oil bath. The systems were cooled to r.t. and neutralized very carefully by concentrated ammonia solution (30%). The mixtures strongly heated themselves during the neutralization so ammonia solution was added very slowly. Crude 2-/3-alkoxyanilines were extracted into 3 × 250 mL diethyl ether (DEE). The organic layer was dried over anhydrous sodium carbonate, filtered and solvent was removed *in vacuo*. Desired 2-/3-alkoxyanilines (**4´a–h**) were purified using vacuum distillation [27]. Yields (in percentages), melting point values as well as IR spectra of the compounds **4´a–h** were published in a paper [27].

General Procedure For the Preparation of 1-Alkoxy-2-/3-isocyanatobenzenes

Anhydrous toluene (1000 mL) was saturated by phosgene (6h) in 3L flask equipped with three ground joints. 2-/3-Alkoxyanilines (**4′a–h**; 1.00 mol), i.e., **4′a**, **4′e** (165.23 g), **4′b**, **4′f** (179.26 g), **4′c**, **4′g** (193.29 g), **4′d** and **4′h** (207.31 g), respectively, were dissolved in anhydrous toluene (200 mL) and added continuously into the saturated solution of phosgene. Particular systems were heated up to reflux (3h) in an oil bath. Finally, solvent was removed *in vacuo* and synthesized 1-alkoxy-2-/3--isocyanatobenzenes (**5′a–h**; Scheme) were purified by vacuum distillation [27].

Yields (in percentages), boiling point values as well as IR spectra of the compounds **4′a–h** were published in a paper [27].

General Procedure For the Preparation of (±)-N-(Oxiran-2-ylmethyl)-N-propylpropanamine

Into an aqueous solution of *N*-propylpropanamine (**6′**; CAS Registry Number 142-84-7; 303.57 g, 3.00 mol), a (±)-2-(chloromethyl)oxirane reagent (CAS Registry Number 106-89-8; 277.56 g, 3.00 mol) was added under vigorous stirring (Scheme). Temperature of the reaction was maintained at 35 °C (2h); solid carbon dioxide in acetone was used for occasional cooling of the system if needed. After passing this procedure, the solution was allowed to stand 48h (r.t.).

A partially crystallized reaction mixture was heated to 75 °C and treated (15 min) with an aqueous sodium hydroxide solution (38%). After cooling to r.t., the solution was filtered and crude intermediate was formed. Continuous extraction of the filtrate with 3 × 250 mL DEE, collecting of all organic fractions, drying over magnesium sulfate and removal of solvent *in vacuo* led to a crude intermediate [28]. Isolation of this product and its crystallization from anhydrous EtOH provided (±)-*N*-(oxiran-2-ylmethyl)-*N*-propylpropanamine (**7′**).

Spectral (IR) and physicochemical (melting point, refractive index n_D) properties as well as elemental analyses results (% C, H, N) of this intermediate confirmed its identity and were already published [28].

General Procedure For the Preparation of 1-(Dipropylamino)-3-pyrrolidin-1-ylpropan-2-ol and 1-Azepan-1--yl-3-(dipropylamino)-propan-2-ol

Addition of a cyclic secondary amine (0.20 mol), i.e., pyrrolidine (CAS Registry Number 123-75-1; 14.22 g) or azepane (hexamethyleneimine; CAS Registry Number 111-49-9; 19.84 g), to a synthesized (±)-*N*-(oxiran-2-ylmethyl)-*N*-propylpropanamine (**7′**; 31.45 g, 0.20 mol) in anhydrous 2-PrOH (150 mL) under reflux (6h) provided crude 1-(dipropylamino)-3-pyrrolidin-1-ylpropan-2-ol (**8′a**) or 1-azepan-1-yl-3-(dipropylamino)-propan-2-ol (**8′b**; Scheme). These intermediates were isolated and dissolved in chloroform. The organic fraction was dried over anhydrous magnesium sulfate, filtered and solvent was removed *in vacuo*.

Final liquid compounds **8′a** and **8′b** were purified by vacuum distillation [29] and some of their spectral (IR) and physicochemical characteristics (boiling point values) were published [21].

General Procedure For the Preparation of 1-(1-Azacycloalkyl)-3-(dipropylamino)propan-2-yl (2-/3-alkoxy-phenyl)carbamates

1-(1-Azacycloalkyl)-3-(dipropylamino)propan-2-yl (2-/3-alkoxyphenyl)carbamates (**9′a–p**; azacycloalkyl = pyrrolidinyl or azepanyl) were synthesized by a reaction of 1-alkoxy-2-/3-iso-cyanatobenzenes (**5′a–h**; 0.20 mol), i.e., **5′a**, **5′e** (38.25 g), **5′b**, **5′f** (41.05 g), **5′c**, **5′g** (43.86 g), **5′d** and **5′h** (46.66 g), respectively, with a dibasic alcohol **8′a** (45.67 g, 0.20 mol) or **8′b** (51.29 g, 0.20 mol) in anhydrous toluene (150 mL) under reflux (8h). After cooling the systems to r.t., crude compounds were isolated, dissolved in chloroform and washed with water. The organic fraction was isolated, dried over anhydrous magnesium sulfate, filtered and solvent was removed *in vacuo*. Crude products were purified by vacuum distillation [21].

Chemical structures of desired bases **9′a–p** were confirmed by spectral analyses (IR). In addition, elemental analyses results (% C, H, N) were within ±0.40% of theoretical values for all proposed molecules [21].

Current liquid chromatography high resolution mass spectroscopy (HPLC-HR-MS) analyses of the compounds **9′a–p** were performed on a chromatographic apparatus consisting of the LC Agilent Infinity System (Agilent Technologies, Santa Clara, CA, USA) equipped with an gradient pump (1290 Bin Pump VL), automatic injector (1260 HiPals), and column thermostat (1290 TCC). The LC system was coupled with the Quadrupole Time-Of-Flight mass spectrometer (6520 Accurate Mass Q-TOF LC/MS). Q-TOF was equipped with an electrospray ionization source operated in a positive and negative ionization mode as well.

For data acquisition and processing, a personal computer with the Mass Hunter software *ver*. MassHunter Workstation B 04.00 (Agilent Technologies) was used. More detailed specifications were provided in a main text of the article. The HPLC-HR-MS characterization of the compounds **9′a–p** is given below.

**Entry**	**Summary Formula (M)**	**$[M + H]^+$ Adduct**			**$[M – H]^-$ Adduct**		
		**Theoretical *m/z***	**Measured *m/z***	**Difference (ppm)**	**Theoretical *m/z***	**Measured *m/z***	**Difference (ppm)**
**9′a**	$C_{24}H_{41}N_3O_3$	420.3221	420.3227	-1.43	418.3075	418.3080	-1.15
**9′b**	$C_{25}H_{43}N_3O_3$	434.3377	434.3388	-2.53	432.3232	432.3230	0.46
**9′c**	$C_{26}H_{45}N_3O_3$	448.3534	448.3537	-0.67	446.3388	446.3381	1.57
**9′d**	$C_{27}H_{47}N_3O_3$	462.3690	462.3682	1.73	460.3545	460.3552	-1.52
**9′e**	$C_{24}H_{41}N_3O_3$	420.3221	420.3231	-2.45	418.3075	418.3081	-1.30
**9′f**	$C_{25}H_{43}N_3O_3$	434.3377	434.3389	-2.66	432.3232	432.3228	0.93
**9′g**	$C_{26}H_{45}N_3O_3$	448.3534	448.3548	-3.13	446.3388	446.3379	2.10
**9′h**	$C_{27}H_{47}N_3O_3$	462.3690	462.3701	-2.44	460.3545	460.3536	1.96
**9′i**	$C_{26}H_{45}N_3O_3$	448.3534	448.3530	0.89	446.3388	446.3390	-0.45
**9′j**	$C_{27}H_{47}N_3O_3$	462.3690	462.3701	-2.38	460.3545	460.3536	1.96
**9′k**	$C_{28}H_{49}N_3O_3$	476.3847	476.3840	1.47	474.3701	474.3694	1.48
**9′l**	$C_{29}H_{51}N_3O_3$	490.4003	490.3998	1.02	488.3858	488.3861	-0.57
**9′m**	$C_{26}H_{45}N_3O_3$	448.3534	448.3547	-3.02	446.3388	446.3389	-0.08
**9′n**	$C_{27}H_{47}N_3O_3$	462.3690	462.3692	-0.52	460.3545	460.3559	-3.04
**9′o**	$C_{28}H_{49}N_3O_3$	476.3847	476.3853	-1.26	474.3701	474.3694	1.56
**9′p**	$C_{29}H_{51}N_3O_3$	490.4003	490.4011	-1.63	488.3858	488.3856	0.26

General Procedure For the Preparation of 1-[2-[({[2-/3-(Alkoxy)phenyl]amino}carbonyl)oxy]-3-(dipropylammonio)propyl]pyrrolidinium oxalates/dichlorides and 1-[2-[({[2-/3-(Alkoxy)phenyl]amino}carbonyl)oxy]-3-(dipropylammonio)propyl]azepanium oxalates/dichlorides

The solutions of particular bases **9′a–p** (0.20 mol) in chloroform (100 mL) were treated with a saturated solution of oxalic acid in anhydrous EtOH or ethereal hydrogen chloride and slowly stirred (5h, r.t.). The solvents were removed *in vacuo* and solid crude products 1-[2-[({[2-/3-(alkoxy)phenyl]amino}carbonyl)oxy]-3-(dipropylammonio)propyl]pyrrolidinium oxalates (**1a–d**)/dichlorides (**1e–h**) as well as 1-[2-[({[2-/3-(alkoxy)phenyl]amino}carbonyl)oxy]-3-(dipropylammonio)-propyl]azepanium oxalates (**1i–l**)/dichlorides (**1m–p**; Scheme, Table 1) were crystallized from acetone (**1i–l**) or mixture of acetone/ethanol (**1a–d**, **1e–h**, **1m–p**). The compounds **1a–p** were achieved with 44% (**1e**) to 76% (**1d**) yields [21].

Chemical structures of synthesized oxalates and dichlorides were verified by interpretation of their IR. In addition, elemental analyses results (% C, H, N) were within ±0.40% of theoretical values for all proposed salts [21].

Physicochemical Properties of Analyzed Compounds

Purity of the molecules **1a–p** was verified by thin-layer chromatography (TLC) using ethanol/benzene/diethyl amine eluant (10:3:0.2, *v*/*v*) as a mobile phase. Spots were observed under iodine vapors/UV light at a wavelength (λ) of 254 nm [21]. Elongation of an alkoxy side chain *R* led to higher R_f values within particular subsets **1a–d**, **1e–h**, **1i–l** and **1m–p**, as expected (Table S1).

All investigated salts **1a–p** were freely soluble in distilled water, soluble in anhydrous EtOH and practically insoluble in chloroform [26]. Their uncorrected melting point values were published in [21] and are provided in Table S1.

Acid-base dissociation constant (pK_{a1}, pK_{a2}) values of analyzed substances **1i–p** were estimated by alkalimetric titration with potentiometric indication of a titration (equivalence) point at 21 °C in an investigated p*H* range from 3.50 to 11.50 [26].

In accordance with knowledge about physicochemical properties of these derivatives, firstly protonization of an aliphatic amine (dipropylamino group) proceeded followed by protonization of a cyclic amine (azepan-1-yl fragment). Conversely, the dissociation constants were assigned to centers of protonation as follows: pK_{a1} for an azepanium moiety and pK_{a2} for a dipropylammonium fragment, respectively. Both pK_{a1} and pK_{a2} values of the derivatives **1i–l** were lower than those of **1m–p**. On the other hand, the pK_{a1} and pK_{a2} constants have not been observed for a subset **1a–h**.

Local Anesthetic Activity of Analyzed Compounds

Relative surface local anesthetic activity ($RLAA_s$; rabbit cornea; 0.01 M cocaine as a standard drug) and infiltration local anesthetic activity ($RLAA_i$; guinea pig; an intradermal application; 0.02 M procaine as a standard drug) of investigated compounds **1a–p** was already published [21].

Descriptors, which defined their relative surface (U_s) as well as infiltration (U_i) local anesthetic efficiency, are listed in Table S1. These parameters were calculated from observed molar concentrations, which provided same local anesthetic effect as a standard, i.e., cocaine or procaine.

As can be seen, the biological screening aimed especially estimation of the U_s indices. In fact, the U_i parameters were observed only for the compound **1b**, **1f**, **1j** and **1n**, respectively. Other molecules have not been tested due to capacity reasons.

It was found that all screened dibasic derivatives of 2-/3-alkoxyphenylcarbamic acid were more effective LAs than cocaine ($RLAA_s$) or procaine ($RLAA_i$) [21].

Acute Toxicity of Analyzed Compounds

Acute toxicity of all compounds **1a–p** was defined by LD_{50} values (in mg/kg units). The LD_{50} descriptor (lethal dose) was the amount of a substance, given all at once, which led to death of 50% (one half) of a group of tested animals (white mice; subcutaneous application). The LD_{50} was considered one way to measure the short-term poisoning potential (acute toxicity) of analyzed derivatives.

The LD_{50} values of the molecules **1a–p** [21] were higher compared to those of cocaine (LD_{50} = 125 mg/kg) indicating lower toxicity of **1a–p** (Table S1). In addition, the molecule **1c** showed identical acute toxicity [21] than procaine (LD_{50} = 600 mg/kg).

Table S1. Chemical structure of presently evaluated compounds **1a**–**p**, their yields (in percentages), molecular formula, molecular weight (MW), melting point (m.p.) values, R_f parameters (TLC) and dissociation constants (pK_{a1}, pK_{a2}) as well as indices describing their relative surface (U_s) and infiltration (U_i) local anesthetic efficiency, respectively. The compounds **1a**–**p** showed relatively low acute toxicity, which was proven by estimated LD_{50} parameters (in mg/kg units).

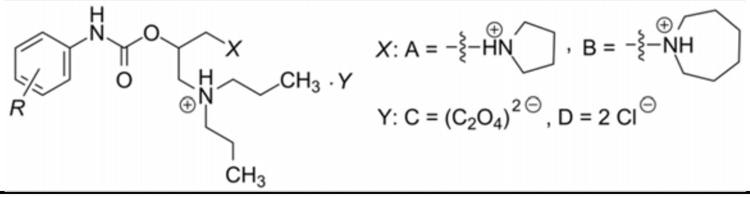

Comp.	*R*	*X*	*Y*	Formula	MW	Yield (%)	m.p.	[3] R_f	pK_{a1}	pK_{a2}	U_s	U_i	LD_{50} (mg/kg)
**1a**	2-OC_4H_9	A	C	$C_{26}H_{43}O_7N_3$	509.64	56	122–123	0.45	[4] –	–	7	[5] *nt*	100–300
**1b**	2-OC_5H_{11}	A	C	$C_{27}H_{45}O_7N_3$	523.66	49	118–120	0.49	–	–	25	40	300
**1c**	2-OC_6H_{13}	A	C	$C_{28}H_{47}O_7N_3$	537.68	67	114–115	0.52	–	–	50	*nt*	600
**1d**	2-OC_7H_{15}	A	C	$C_{29}H_{49}O_7N_3$	551.71	76	129–130	0.55	–	–	10	*nt*	300
**1e**	3-OC_4H_9	A	D	$C_{24}H_{43}O_3Cl_2N_3$	492.52	44	184–185	0.54	–	–	80	*nt*	100
**1f**	3-OC_5H_{11}	A	D	$C_{25}H_{45}O_3Cl_2N_3$	506.55	56	167–168	0.58	–	–	130	250	200–250
**1g**	3-OC_6H_{13}	A	D	$C_{26}H_{47}O_3Cl_2N_3$	520.58	45	145–147	0.63	–	–	50	*nt*	400
**1h**	3-OC_7H_{15}	A	D	$C_{27}H_{49}O_3Cl_2N_3$	534.60	65	143–1146	0.66	–	–	5	*nt*	100–300
**1i**	2-OC_4H_9	B	C	$C_{28}H_{47}O_7N_3$	537.69	56	90–92	0.62	6.50	8.22	8	*nt*	400–500
**1j**	2-OC_5H_{11}	B	C	$C_{29}H_{49}O_7N_3$	551.71	67	108–110	0.65	6.37	8.18	3	6	300–450
**1k**	2-OC_6H_{13}	B	C	$C_{30}H_{51}O_7N_3$	565.74	52	103–106	0.69	6.30	8.09	*nt*	*nt*	200–600
**1l**	2-OC_7H_{15}	B	C	$C_{31}H_{53}O_7N_3$	579.77	49	98–100	0.72	6.18	8.01	*nt*	*nt*	400–500
**1m**	3-OC_4H_9	B	D	$C_{26}H_{47}O_3Cl_2N_3$	520.58	57	144–146	0.65	6.70	8.32	100	*nt*	300–350
**1n**	3-OC_5H_{11}	B	D	$C_{27}H_{49}O_3Cl_2N_3$	534.60	54	131–133	0.68	6.73	8.24	50	100	200–400
**1o**	3-OC_6H_{13}	B	D	$C_{28}H_{51}O_3Cl_2N_3$	548.63	63	170–173	0.72	6.35	8.15	8	*nt*	150–450
**1p**	3-OC_7H_{15}	B	D	$C_{29}H_{53}O_3Cl_2N_3$	562.66	62	158–160	0.74	6.24	8.12	*nt*	*nt*	100–300
[1] **Cocaine**											1	3.6	125
[2] **Procaine**											0.1	1	600

[1] Cocaine, [2] Procaine, reference local anesthetic drugs used for evaluation of relative surface (U_s) and infiltration (U_i) local anesthetic activity of drug candidates; [3] R_f, retardation factor observed by thin-layer chromatography (TLC) when using ethanol/benzene/diethyl amine as eluant (10:3:0.2, *v*/*v*); [4] –, values have not been estimated; [5] *nt*, a compound has not been tested.

Table S2. Relationships between number of carbon atoms forming the alkoxy side chain *R* (n_c; alkoxy = butoxy to heptyloxy) and γ values (in N/m units) of evaluated compounds **1a–p**. The relationships were expressed by polynomial functions of 2nd order (Equations (S1)–(S4); Eqs.) and characterized by values of relevant statistical descriptors, i.e., number of points (number of cases; *n*), degrees of freedom (*DF*), reduced chi-square (χ^2_{red}), residual sum of squares (*RSS*), correlation coefficient (*R*), adjusted coefficient of determination (*Adj. R*^{2}), root mean squared error (standard deviation; *RMSE*), norm of residuals (*NR*), Fisher´s significance ratio (Fisher´s *F*-test; *F*) and probability of obtaining the *F* Ratio (significance of a whole model; *Prob > F*), respectively.

Equation No.	Series	Equation	Statistical Descriptors
**Eq. (S1)**	**1a–d**	$\gamma = -0.0003\ (\pm0.0001) \times n_c^2 + 0.0022\ (\pm0.0006) \times n_c + 0.06121\ (\pm0.0016)$	$n = 4$, $DF = 1$, $\chi^2_{red} = 1.104 \times 10^{-8}$, $RSS = 1.104 \times 10^{-8}$, $R = 0.9996$, $Adj.\ R^2 = 0.9975$, $RMSE = 1.051 \times 10^{-4}$, $NR = 1.051 \times 10^{-4}$, $F = 587.63$, [1] $Prob > F = 0.02916$ *
**Eq. (S2)**	**1e–h**	$\gamma = -0.0007\ (\pm0.0001) \times n_c^2 + 0.0062\ (\pm0.001) \times n_c + 0.0501\ (\pm0.0003)$	$n = 4$, $DF = 1$, $\chi^2_{red} = 5.000 \times 10^{-10}$, $RSS = 5.000 \times 10^{-10}$, $R = 0.9999$, $Adj.\ R^2 = 0.9999$, $RMSE = 2.236 \times 10^{-5}$, $NR = 2.236 \times 10^{-5}$, $F = 17738.12$, $Prob > F = 0.0053$ **
**Eq. (S3)**	**1i–l**	$\gamma = 0.0003\ (\pm0.0001) \times n_c^2 + 0.0017\ (\pm0.0003) \times n_c + 0.0608\ (\pm0.0009)$	$n = 4$, $DF = 1$, $\chi^2_{red} = 3.380 \times 10^{-9}$, $RSS = 3.380 \times 10^{-9}$, $R = 0.9999$, $Adj.\ R^2 = 0.9991$, $RMSE = 5.814 \times 10^{-5}$, $NR = 5.814 \times 10^{-5}$, $F = 1687.44$, $Prob > F = 0.0172$ *
**Eq. (S4)**	**1m–p**	$\gamma = -0.0002\ (\pm0.0001) \times n_c^2 + 0.0004\ (\pm0.0010) \times n_c + 0.0650\ (\pm0.0027)$	$n = 4$, $DF = 1$, $\chi^2_{red} = 3.281 \times 10^{-8}$, $RSS = 3.281 \times 10^{-8}$, $R = 0.9992$, $Adj.\ R^2 = 0.9954$, $RMSE = 1.811 \times 10^{-4}$, $NR = 1.811 \times 10^{-4}$, $F = 322.35$, $Prob > F = 0.0394$ *

[1], The indication of a significance level of the *F* Ratio: * (one star), statistically significant; ** (two stars), statistically very significant.

Table S3. Purity (in percentages) and values of logarithms of retention (capacity) factors (log *k*) from RP-HPLC of investigated compounds **1a–p**. The log *k* values were determined in methanol (MeOH)/water mobile phases containing a varying volume ratio (*v*/*v*) of the organic modifier.

Comp.	[1] Purity (%)	Mobile phase MeOH/water (*v*/*v*)				
		k; 80:20	*k*; 85:15	*k*; 90:10	*k*; 95:5	*k*; pure MeOH
**1a**	97.84	5.2060	3.7636	2.4877	1.5878	0.9114
**1b**	97.27	6.6904	4.6302	3.2092	1.9235	1.0479
**1c**	97.49	10.4858	6.5494	4.0300	2.3351	1.3449
**1d**	97.86	12.8588	7.7304	5.0373	2.5421	1.4365
**1e**	97.64	3.6174	2.0811	1.4054	0.7155	0.5063
**1f**	99.29	4.5436	3.0981	1.7972	0.8052	0.5113
**1g**	98.35	6.6896	4.0776	2.3329	1.3128	0.6213
**1h**	97.55	8.9557	5.1988	2.8609	1.6188	0.6091
**1i**	97.17	9.7297	6.5358	4.2073	2.4854	1.3198
**1j**	96.82	12.1983	7.9086	4.8173	2.8242	1.3960
**1k**	96.88	20.0586	11.0255	6.3650	3.3274	2.1394
**1l**	97.26	30.4159	16.4135	9.1601	4.2374	2.5096
**1m**	99.65	6.0325	3.1060	1.8599	1.1026	0.5922
**1n**	99.14	8.9166	4.5102	2.6333	1.2419	0.7858
**1o**	97.86	12.0420	5.0804	3.2915	1.2868	0.8674
**1p**	97.59	14.0734	6.6298	4.0476	1.6036	0.7759

[1], Purity (%), purity of the compounds **1a–p** estimated by RP-HPLC using 90% MeOH (*v*/*v*) as a mobile phase.

Table S4. Relationships between number of carbon atoms forming the alkoxy side chain *R* (n_c; alkoxy = butoxy to heptyloxy) and log k_w values (RP-HPLC) of evaluated compounds **1a**–**p**. The relationships were expressed by linear functions (Equations (S5)–(S8); Eqs.) and characterized by values of relevant statistical descriptors, i.e., number of points (number of cases; *n*), degrees of freedom (*DF*), reduced chi-square (χ^2_{red}), residual sum of squares (*RSS*), correlation coefficient (*R*), adjusted coefficient of determination (*Adj.* R^2), root mean squared error (standard deviation; *RMSE*), norm of residuals (*NR*), Fisher´s significance ratio (Fisher´s *F*-test; *F*) and probability of obtaining the *F* Ratio (significance of a whole model; *Prob > F*), respectively.

Equation No.	Series	Equation	Statistical Descriptors
**Eq. (S5)**	**1a–d**	$\log k_w = 0.4099 (\pm 0.0368) \times n_c + 2.0874 (\pm 0.2067)$	*n* = 4, *DF* = 2, χ^2_{red} = 0.0068, *RSS* = 0.0136, *R* = 0.9920, *Adj.* R^2 = 0.9762, *RMSE* = 0.0823, *NR* = 0.1164, *F* = 123.93, [1] *Prob > F* = 0.0080 **
**Eq. (S6)**	**1e–h**	$\log k_w = 0.4810 (\pm 0.0499) \times n_c + 2.1496 (\pm 0.2799)$	*n* = 4, *DF* = 2, χ^2_{red} = 0.0124, *RSS* = 0.0249, *R* = 0.9894, *Adj.* R^2 = 0.9684, *RMSE* = 0.1115, *NR* = 0.1577, *F* = 93.03, *Prob > F* = 0.0106 *
**Eq. (S7)**	**1i–l**	$\log k_w = 0.4675 (\pm 0.0483) \times n_c + 2.5403 (\pm 0.2708)$	*n* = 4, *DF* = 2, χ^2_{red} = 0.0116, *RSS* = 0.0233, *R* = 0.9895, *Adj.* R^2 = 0.9687, *RMSE* = 0.1079, *NR* = 0.1526, *F* = 93.89, *Prob > F* = 0.0105 *
**Eq. (S8)**	**1m–p**	$\log k_w = 0.4843 (\pm 0.0090) \times n_c + 2.7738 (\pm 0.0507)$	*n* = 4, *DF* = 2, χ^2_{red} = 4.085×10^{-4}, *RSS* = 8.170×10^{-4}, *R* = 0.9997, *Adj.* R^2 = 0.9990, *RMSE* = 0.0202, *NR* = 0.0286, *F* = 2871.18, *Prob > F* = 0.0004 ***

[1], The indication of a significance level of the *F* Ratio: * (one star), statistically significant; ** (two stars), statistically very significant; *** (three stars), statistically extremely significant.

Table S5. Relationships between the slope (*S*) and log k_w values (RP-HPLC) of evaluated compounds **1a**–**p**. The relationships were expressed by linear functions (Equations (S9)–(S12); Eqs.) and values of relevant statistical descriptors, i.e., number of points (number of cases; *n*), degrees of freedom (*DF*), reduced chi-square (χ^2_{red}), residual sum of squares (*RSS*), correlation coefficient (*R*), adjusted coefficient of determination (*Adj.* R^2), root mean squared error (standard deviation; *RMSE*), norm of residuals (*NR*), Fisher´s significance ratio (Fisher´s *F*-test; *F*) and probability of obtaining the *F* Ratio (significance of a whole model; *Prob > F*), respectively.

Equation No.	Series	Equation	Statistical Descriptors
**Eq. (S9)**	**1a–d, 1i–l**	$S = 0.8172 (\pm 0.0154) \times \log k_w + 0.6883 (\pm 0.0735)$	*n* = 8, *DF* = 6, χ^2_{red} = 0.0008, *RSS* = 0.0045, *R* = 0.9989, *Adj.* R^2 = 0.9975, *RMSE* = 0.0274, *NR* = 0.0671, *F* = 2807.28, [1] *Prob > F* = 0.0001 ***
**Eq. (S10)**	**1e–h, 1m–p**	$S = 0.8845 (\pm 0.0202) \times \log k_w + 0.7755 (\pm 0.1042)$	*n* = 8, *DF* = 6, χ^2_{red} = 0.0013, *RSS* = 0.0078, *R* = 0.9984, *Adj.* R^2 = 0.9964, *RMSE* = 0.0360, *NR* = 0.0883, *F* = 1915.62, *Prob > F* = 0.0001 ***
**Eq. (S11)**	**1a–h**	$S = 1.0065 (\pm 0.1311) \times \log k_w + 0.0396 (\pm 0.6032)$	*n* = 8, *DF* = 6, χ^2_{red} = 0.0420, *RSS* = 0.2522, *R* = 0.9527, *Adj.* R^2 = 0.8922, *RMSE* = 0.2050, *NR* = 0.5022, *F* = 58.95, *Prob > F* = 0.0002 ***
**Eq. (S12)**	**1i–p**	$S = 0.9874 (\pm 0.1547) \times \log k_w + 0.0057 (\pm 0.8207)$	*n* = 8, *DF* = 6, χ^2_{red} = 0.0599, *RSS* = 0.3595, *R* = 0.9336, *Adj.* R^2 = 0.8502, *RMSE* = 0.2448, *NR* = 0.5996, *F* = 40.72, *Prob > F* = 0.0007 ***

[1], The indication of a significance level of the *F* Ratio: * (one star), statistically significant; ** (two stars), statistically very significant; *** (three stars), statistically extremely significant.

Table S6. Relationships between the log k_w values (RP-HPLC) and *in silico* log *P* parameters of evaluated compounds **1a–p** and non-protonated bases **9′a–p**. The relationships were expressed by linear functions (Equations (S13)–(S24); Eqs.) and characterized by values of common statistical descriptors, i.e., number of points (number of cases; *n*), degrees of freedom (*DF*), reduced chi-square (χ^2_{red}), residual sum of squares (*RSS*), correlation coefficient (*R*), adjusted coefficient of determination (*Adj. R*²), root mean squared error (standard deviation; *RMSE*), norm of residuals (*NR*), Fisher′s significance ratio (Fisher′s *F*-test; *F*) and probability of obtaining the *F* Ratio (significance of a whole model; *Prob* > *F*), respectively.

Equation No.	Series	Equation	Statistical Descriptors
**Eq. (S13)**	**1i–p/ 9′a–p**	log k_w = 0.9893 (±0.0961) × log P_{Cr} – 0.6479 (±0.5443)	*n* = 16, *DF* = 14, χ^2_{red} = 0.0579, *RSS* = 0.8099, *R* = 0.9399, *Adj. R*² = 0.8750, *RMSE* = 0.2405, *NR* = 0.9000, *F* = 106.02, [1] *Prob* > *F* = 0.0001 ***
**Eq. (S14)**	**1i–p/ 9′a–p**	log k_w = 1.0415 (±0.1010) × log P_V – 0.7451 (±0.5528)	*n* = 16, *DF* = 14, χ^2_{red} = 0.0577, *RSS* = 0.8077, *R* = 0.9400, *Adj. R*² = 0.8754, *RMSE* = 0.2402, *NR* = 0.8987, *F* = 106.34, *Prob* > *F* = 0.0001 ***
**Eq. (S15)**	**1i–p/ 9′a–p**	log k_w = 0.9065 (±0.0877) × log P_B – 0.1998 (±0.4990)	*n* = 16, *DF* = 14, χ^2_{red} = 0.0574, *RSS* = 0.8042, *R* = 0.9403, *Adj. R*² = 0.8759, *RMSE* = 0.2397, *NR* = 0.8967, *F* = 106.87, *Prob* > *F* = 0.0001 ***
**Eq. (S16)**	**1i–p/ 9′a–p**	log k_w = 0.7566 (±0.0758) × CLOGP 4.0 – 0.8170 (±0.5781)	*n* = 16, *DF* = 14, χ^2_{red} = 0.0611, *RSS* = 0.8550, *R* = 0.9364, *Adj. R*² = 0.8681, *RMSE* = 0.2471, *NR* = 0.9247, *F* = 99.69, *Prob* > *F* = 0.0001 ***
**Eq. (S17)**	**1i–p/ 9′a–p**	log k_w = 0.8500 (±0.0791) × XLOGP 2.0 + 0.0471 (±0.4572)	*n* = 16, *DF* = 14, χ^2_{red} = 0.0536, *RSS* = 0.7506, *R* = 0.9444, *Adj. R*² = 0.8842, *RMSE* = 0.2315, *NR* = 0.8664, *F* = 115.50, *Prob* > *F* = 0.0001 ***
**Eq. (S18)**	**1i–p/ 9′a–p**	log k_w = 0.8987 (±0.0825) × XLOGP 3.0 – 0.7388 (±0.5227)	*n* = 16, *DF* = 14, χ^2_{red} = 0.0523, *RSS* = 0.7326, *R* = 0.9458, *Adj. R*² = 0.8869, *RMSE* = 0.2288, *NR* = 0.8559, *F* = 118.68, *Prob* > *F* = 0.0001 ***
**Eq. (S19)**	**1i–p/ 9′a–p**	log k_w = 2.0498 (±0.1988) × MLOGP – 1.7580 (±0.6506)	*n* = 16, *DF* = 14, χ^2_{red} = 0.0577, *RSS* = 0.8079, *R* = 0.9400, *Adj. R*² = 0.8753, *RMSE* = 0.2402, *NR* = 0.8989, *F* = 106.31, *Prob* > *F* = 0.0001 ***
**Eq. (S20)**	**1i–p/ 9′a–p**	log k_w = 1.0233 (±0.0931) × ACLOGP – 1.2887 (±0.5681)	*n* = 16, *DF* = 14, χ^2_{red} = 0.0515, *RSS* = 0.7215, *R* = 0.9466, *Adj. R*² = 0.8887, *RMSE* = 0.2270, *NR* = 0.8494, *F* = 120.72, *Prob* > *F* = 0.0001 ***
**Eq. (S21)**	**1i–p/ 9′a–p**	log k_w = 0.8218 (±0.0761) × miLogP 2.2 – 0.4803 (±0.5035)	*n* = 16, *DF* = 14, χ^2_{red} = 0.0531, *RSS* = 0.7440, *R* = 0.9449, *Adj. R*² = 0.8852, *RMSE* = 0.2305, *NR* = 0.8626, *F* = 116.64, *Prob* > *F* = 0.0001 ***
**Eq. (S22)**	**1i–p/ 9′a–p**	log k_w = 0.9046 (±0.0876) × ALOGP – 1.0117 (±0.5779)	*n* = 16, *DF* = 14, χ^2_{red} = 0.0576, *RSS* = 0.8062, *R* = 0.9402, *Adj. R*² = 0.8756, *RMSE* = 0.2400, *NR* = 0.8979, *F* = 106.56, *Prob* > *F* = 0.0001 ***
**Eq. (S23)**	**1i–p/ 9′a–p**	log k_w = 1.1602 (±0.1091) × log P_{S-IT} – 1.0565 (±0.5654)	*n* = 16, *DF* = 14, χ^2_{red} = 0.0547, *RSS* = 0.7652, *R* = 0.9433, *Adj. R*² = 0.8819, *RMSE* = 0.2338, *NR* = 0.8748, *F* = 113.02, *Prob* > *F* = 0.0001 ***
**Eq. (S24)**	**1i–p/ 9′a–p**	log k_w = 1.2830 (±0.1172) × ALOGPs 2.1 – 2.8688 (±0.7141)	*n* = 16, *DF* = 14, χ^2_{red} = 0.0519, *RSS* = 0.7265, *R* = 0.9462, *Adj. R*² = 0.8879, *RMSE* = 0.2278, *NR* = 0.8524, *F* = 119.79, *Prob* > *F* = 0.0001 ***

[1], The indication of a significance level of the *F* Ratio: *** (three stars), statistically extremely significant.

Table S7. Relationships between the γ (in N/m units) and log k_w values (RP-HPLC) of evaluated compounds **1a–p**. The relationships were expressed by linear functions (Equations (S25)–(S28); Eqs.) and characterized by values of relevant statistical descriptors, i.e., number of points (number of cases; *n*), degrees of freedom (*DF*), reduced chi-square (χ^2_{red}), residual sum of squares (*RSS*), correlation coefficient (*R*), adjusted coefficient of determination (*Adj. R*^{2}), root mean squared error (standard deviation; *RMSE*), norm of residuals (*NR*), Fisher′s significance ratio (Fisher′s *F*-test; *F*) and probability of obtaining the *F* Ratio (significance of a whole model; *Prob > F*), respectively.

Equation No.	Series	Equation	Statistical Descriptors
**Eq. (S25)**	**1a–d, 1i–l**	$\gamma = -0.0029\ (\pm 0.0004) \times \log k_w + 0.0756\ (\pm 0.0017)$	$n = 8$, $DF = 6$, $\chi^2_{red} = 4.054 \times 10^{-7}$, $RSS = 2.432 \times 10^{-6}$, $R = 0.9573$, $Adj.\ R^2 = 0.9024$, $RMSE = 6.367 \times 10^{-7}$, $NR = 0.0016$, $F = 65.70$, [1] $Prob > F = 0.0002$ ***
**Eq. (S26)**	**1e–h, 1m–p**	$\gamma = -0.0033\ (\pm 0.0006) \times \log k_w + 0.0776\ (\pm 0.0030)$	$n = 8$, $DF = 6$, $\chi^2_{red} = 1.049 \times 10^{-6}$, $RSS = 6.292 \times 10^{-6}$, $R = 0.9204$, $Adj.\ R^2 = 0.8217$, $RMSE = 0.0010$, $NR = 0.0025$, $F = 32.25$, $Prob > F = 0.0012$ **
**Eq. (S27)**	**1a–h**	$\gamma = -0.0036\ (\pm 0.0005) \times \log k_w + 0.0782\ (\pm 0.0022)$	$n = 8$, $DF = 6$, $\chi^2_{red} = 5.806 \times 10^{-7}$, $RSS = 3.484 \times 10^{-6}$, $R = 0.9481$, $Adj.\ R^2 = 0.8820$, $RMSE = 0.0008$, $NR = 0.0019$, $F = 53.32$, $Prob > F = 0.0003$ ***
**Eq. (S28)**	**1i–p**	$\gamma = -0.0036\ (\pm 0.0003) \times \log k_w + 0.0798\ (\pm 0.0014)$	$n = 8$, $DF = 6$, $\chi^2_{red} = 1.798 \times 10^{-7}$, $RSS = 1.079 \times 10^{-6}$, $R = 0.9841$, $Adj.\ R^2 = 0.9632$, $RMSE = 0.0004$, $NR = 0.0010$, $F = 184.13$, $Prob > F = 0.0001$ ***

[1], The indication of a significance level of the *F* Ratio: ** (two stars), statistically very significant; *** (three stars), statistically extremely significant.

Table S8. Squared cosines (cos2) of the variables 1–14. Indication of the variables was as follows: 1 (the loading based on the log (1/*MIC* [M]) values, which were observed after 14-d *in vitro* cultivation against MT_v $H_{37}R_v$), 2 (MT_v $H_{37}R_v$, 21-d), 3 (*MK* 235/80, 7-d), 4 (*MA* 330/80, 14-d), 5 (*MK* 235/80, 21-d), 6 (*MK* 235/80, 14-d), 7 (*MK* 6509/96, 14-d), 8 (*MK* 6509/96, 7-d), 9 (*MA* 330/80, 21-d), 10 (*MK* 6509/96, 21-d), 11 (MT_a $H_{37}R_a$, 7-d), 12 (*MM*, 21-d), 13 (*MK* DSM, 7-d) and 14 (*MS*, 3-d), respectively. Values in grey cells corresponded for each variable to the Principal Component (PC), for which the cos2 was the largest. The first two interpreted PCs of the analysis (with $\lambda_e > 1.0$) accounted for 89.59% of the total variance in the data as follows: 77.22% (PC 1) and 12.37% (PC 2), respectively.

**Variable**	**cos2 ([1] PC 1)**	**cos2 ([2] PC 2)**	**cos2 ([3] PC 3)**
**1**	0.573	0.388	0.002
**2**	0.568	0.296	0.026
**3**	0.763	0.080	0.122
**4**	0.858	0.041	0.006
**5**	0.875	0.005	0.005
**6**	0.920	0.001	0.002
**7**	0.907	0.001	0.011
**8**	0.928	0.001	0.021
**9**	0.937	0.000	0.012
**10**	0.911	0.000	0.006
**11**	0.735	0.124	0.072
**12**	0.702	0.226	0.012
**13**	0.653	0.273	0.014
**14**	0.481	0.295	0.172

[1], PC 1, Principal Component 1; [2], PC 2, Principal Component 2; [3], PC 3, Principal Component 3.

Table S9. Relationships between the independent variable, i.e., γ (in [N/m] units), log $\varepsilon_{2\,(Ch\text{-}T)}$ or log k_w, and *in vitro* activity (in log (1/*MIC* [M]) units) after 3- (3-d), 14- (14-d) or 21-day (21-d) cultivation of the compounds under the study. The relationships were expressed by linear functions or polynomial functions of 2nd order (Equations (S29)–(S32); Eqs.) and characterized by values of relevant statistical descriptors, i.e., number of points (number of cases; *n*), degrees of freedom (*DF*), reduced chi-square (χ^2_{red}), residual sum of squares (*RSS*), correlation coefficient (*R*), adjusted coefficient of determination (*Adj.* R^2), root mean squared error (standard deviation; *RMSE*), norm of residuals (*NR*), Fisher´s significance ratio (Fisher´s *F*-test; *F*) and probability of obtaining the *F* Ratio (significance of a whole model; *Prob* > *F*), respectively.

Equation No.	Strain (Days of Cultivation)/Series	Equation	Statistical Descriptors
**Eq. (S29)**	[1a] ***MT*v H37Rv (14-d)/ 1a–d, 1i–l**	log (1/*MIC* [M]) = 12.9547 (±0.9382) – 130.1329 (±15.1663) × γ	n = 8, DF = 6, χ^2_{red} = 0.0067, RSS = 0.0401, R = 0.9616, *Adj.* R^2 = 0.9121, $RMSE$ = 0.0818, NR = 0.2003, F = 73.62, [1] $Prob > F$ = 0.0001 ***
**Eq. (S30)**	***MT*v H37Rv (14-d)/ 1a–d, 1i–l**	log (1/*MIC* [M]) = 3.0920 (±0.2822) + 0.3844 (±0.0592) × log k_w	n = 8, DF = 6, χ^2_{red} = 0.0111, RSS = 0.0663, R = 0.9357, *Adj.* R^2 = 0.8547, $RMSE$ = 0.1051, NR = 0.2575, F = 42.18, $Prob > F$ = 0.0006 ***
**Eq. (S31)**	***MT*v H37Rv (14-d)/ 1e–h, 1m–p**	log (1/*MIC* [M]) = 3.6297 (±0.4817) + 0.2941 (±0.0934) × log k_w	n = 8, DF = 6, χ^2_{red} = 0.0278, RSS = 0.1666, R = 0.7892, *Adj.* R^2 = 0.5599, $RMSE$ = 0.1667, NR = 0.4082, F = 9.91, $Prob > F$ = 0.0199 *
**Eq. (S32)**	***MT*v H37Rv (21-d)/ 1i–l**	log (1/*MIC* [M]) = –125.4292 (±1.8159 × 10^{-11}) × (log $\varepsilon_{2\,(Ch\text{-}T)}$)^2 + 1041.0621 (±1.5082 × 10^{-10}) × log $\varepsilon_{2\,(Ch\text{-}T)}$ – 2155.0943 (±3.1309 × 10^{-10})	n = 4, DF = 1, χ^2_{red} = 5.120 × 10^{-27}, RSS = 5.120 × 10^{-27}, R = 1.0000, R^2 = 1.0000, *Adj.* R^2 = 1.0000, $RMSE$ = 7.1554 × 10^{-14}, NR = 7.1554 × 10^{-14}, F = 2.43 × 10^{25}, $Prob > F$ = 0.0000 ***
**Eq. (S33)**	[1b] ***MS* (3-d)/ 1c–h**	log (1/*MIC* [M]) = –6.7443 (±1.0328) × (log $\varepsilon_{2\,(Ch\text{-}T)}$)^2 + 57.1838 (±8.8932) × log $\varepsilon_{2\,(Ch\text{-}T)}$ –116.3929 (±19.1277)	n = 6, DF = 3, χ^2_{red} = 0.0028, RSS = 0.0085, R = 0.9806, *Adj.* R^2 = 0.9358, $RMSE$ = 0.0533, NR = 0.0922, F = 37.44, $Prob > F$ = 0.0076 **

[1a] *MT*v H37Rv, *Mycobacterium tuberculosis* CNCTC My 331/88 (*M. tuberculosis* H37Rv); [1b] *MS*, *Mycobacterium smegmatis* ATCC 700084; [1] The indication of a significance level of the *F* Ratio: ** (two stars), statistically very significant; *** (three stars), statistically extremely significant. The relationships (i) γ *versus* log (1/*MIC* [M]) based on 14-d *in vitro* screening of the 3-alkoxy substituted compounds (**1e–h** and **1m–p**) against *MT*v H37Rv (linear model); as well as (ii) log $\varepsilon_{2\,(Ch\text{-}T)}$ *versus* log (1/*MIC* [M]) based on 21-d *in vitro* screening of the 3-alkoxy substituted compounds (**1m–p**) against *MT*v H37Rv (model built on a polynomial function of 2nd order) were statistically insignificant (*Prob* > *F* ≥ 0.0500).

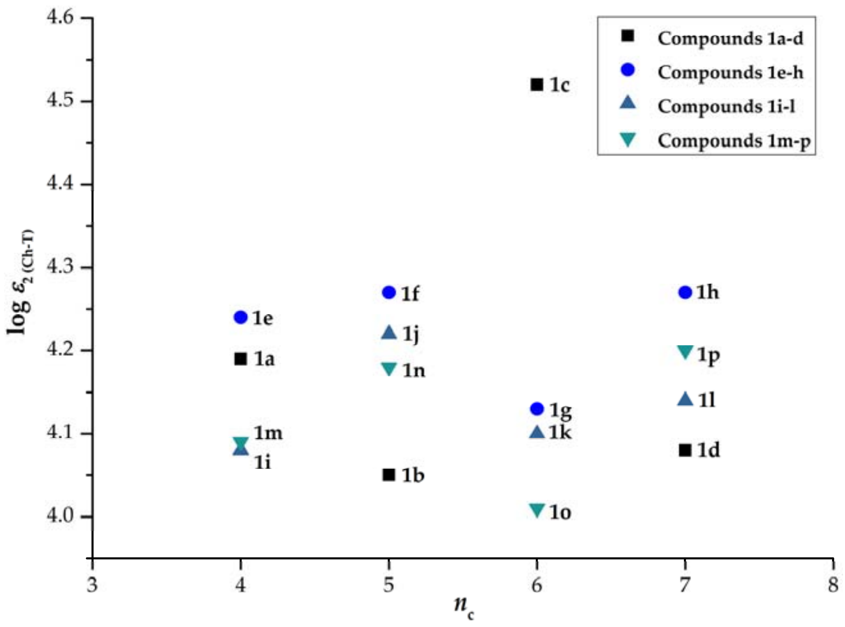

Figure S1. Relationships between number of carbon atoms forming the alkoxy side chain R (n_c; alkoxy = butoxy to heptyloxy) and log $\varepsilon_{2\,(Ch\text{-}T)}$ values of analyzed sets **1a–d**, **1e–h**, **1i–l** and **1m–p**.

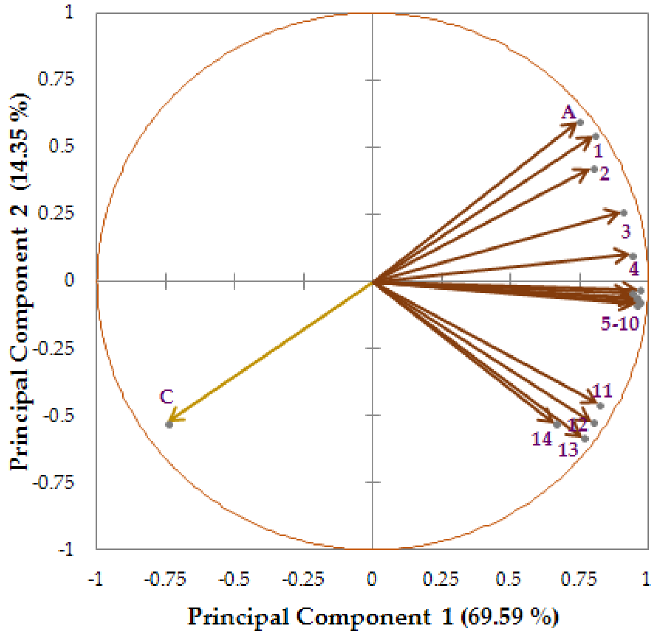

Figure S2. Two dimensional mapping of the loadings of variables (variously colored vectors) indicating their (i) positions towards a circle of correlation; and (ii) relationships to both Principal Component 1 and 2. Indication and numbering of the vectors was as follows: A (the vector related to the log k_w parameter), C (γ), 1 (the vector based on the log (1/*MIC* [M]) values, which were observed after 14-d *in vitro* cultivation against MT_v $H_{37}R_v$), 2 (MT_v $H_{37}R_v$, 21-d), 3 (*MK* 235/80, 7-d), 4 (*MA* 330/80, 14-d), 5 (*MK* 235/80, 21-d), 6 (*MK* 235/80, 14-d), 7 (*MK* 6509/96, 14-d), 8 (*MK* 6509/96, 7-d), 9 (*MA* 330/80, 21-d), 10 (*MK* 6509/96, 21-d), 11 (MT_a $H_{37}R_a$, 7-d), 12 (*MM*, 21-d), 13 (*MK* DSM, 7-d) and 14 (*MS*, 3-d), respectively. A missing vector B (log $\varepsilon_{2\,(Ch\text{-}T)}$) was not sufficiently defined on both Principal Component 1 and 2.

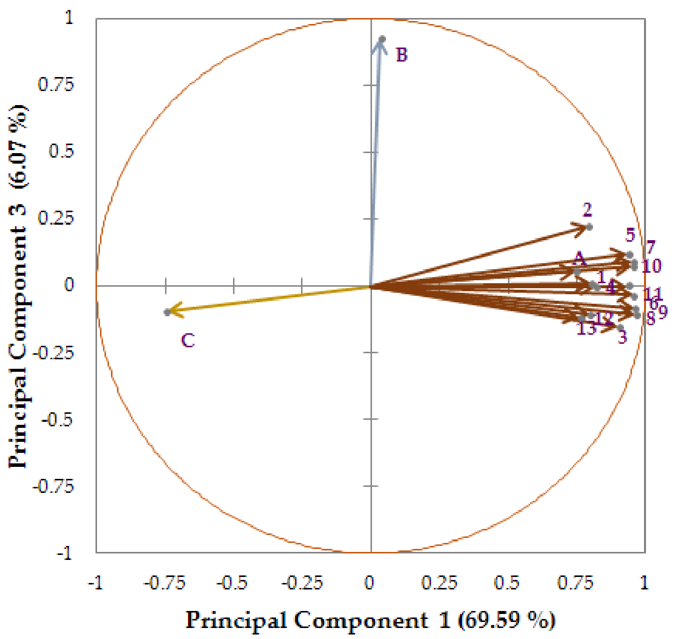

Figure S3. Two dimensional mapping of the loadings of variables (variously colored vectors) indicating their (i) positions towards a circle of correlation; and (ii) relationships to both Principal Component 1 and 3. Indication and numbering of the vectors was as follows: A (the vector related to the log k_w parameter), B (log $\varepsilon_{2\,(Ch\text{-}T)}$), C ($\gamma$), 1 (the vector based on the log (1/*MIC* [M]) values, which were observed after 14-d *in vitro* cultivation against *MT*$_v$ $H_{37}R_v$), 2 (*MT*$_v$ $H_{37}R_v$, 21-d), 3 (*MK* 235/80, 7-d), 4 (*MA* 330/80, 14-d), 5 (*MK* 235/80, 21-d), 6 (*MK* 235/80, 14-d), 7 (*MK* 6509/96, 14-d), 8 (*MK* 6509/96, 7-d), 9 (*MA* 330/80, 21-d), 10 (*MK* 6509/96, 21-d), 11 (*MT*$_a$ $H_{37}R_a$, 7-d), 12 (*MM*, 21-d), 13 (*MK* DSM, 7-d) and 14 (*MS*, 3-d), respectively.

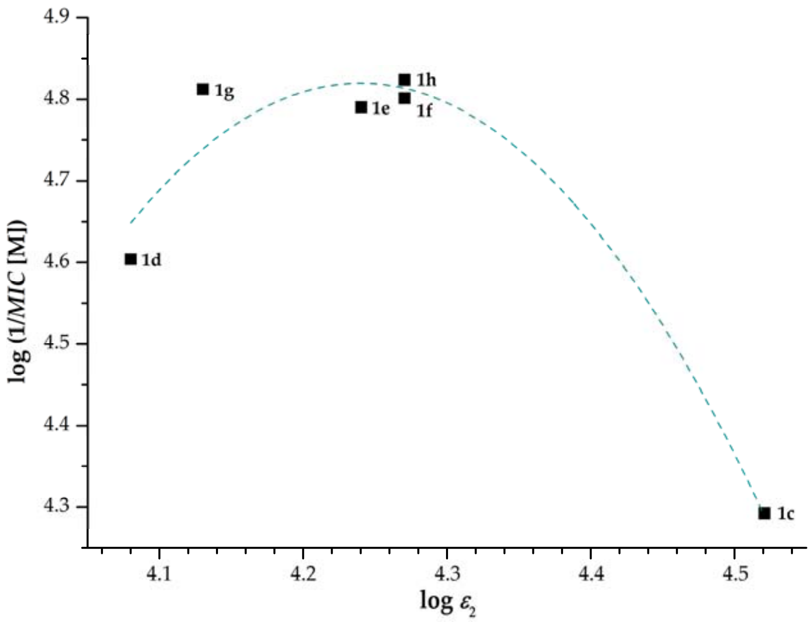

Figure S4. Relationships between the log $\varepsilon_{2\,(Ch\text{-}T)}$ values and log (1/MIC [M]) parameters connected with 3-d *in vitro* screening of the compounds **1c–h** against *Mycobacterium smegmatis* ATCC 700084.
